# Professional-Facing Digital Health Technology for the Care of Patients With Chronic Pain: Scoping Review

**DOI:** 10.2196/66457

**Published:** 2025-05-14

**Authors:** Haruno McCartney, Ashleigh Main, Natalie McFayden Weir, Harleen Kaur Rai, Maryam Ibrar, Roma Maguire

**Affiliations:** 1 Department of Computer and Information Sciences University of Strathclyde Glasgow United Kingdom; 2 Strathclyde Institute of Pharmacy & Biomedical Sciences (Sipbs) University of Strathclyde Glasgow United Kingdom

**Keywords:** chronic pain, digital health, eHealth, clinician, health care professional

## Abstract

**Background:**

Chronic pain is a highly prevalent condition, estimated to affect as many as 30% of people worldwide. The need for more innovative solutions for chronic pain management is clear, and digital health technology (DHT) may be the best way to address this challenge. Much of the digital health research focusing on chronic pain focuses on patient-facing solutions; however, DHT for health care professionals (HCPs) is equally important to support evidence-based practice, which, in turn, improves patient outcomes. Despite this, no review has investigated the availability of professional-facing DHT for chronic pain management.

**Objective:**

This scoping review aims to identify the available professional-facing DHTs for chronic pain management. Specifically, the objectives were to investigate the components of the DHTs as well as development methods, user features, outcomes, and HCP perspectives on DHTs for chronic pain care.

**Methods:**

Databases, including MEDLINE, Embase, CINAHL, PsycINFO, and Inspec, were searched using comprehensive search strategies. Two independent reviewers screened titles and abstracts for inclusion of studies in the review and conducted full-text screening. Any conflicts in each stage of the screening process were first resolved through discussion and then through a third independent reviewer. Data extraction and quality assessment were completed using the Template for Intervention Description and Replication (TiDIER) checklist and Quality Assessment for Diverse Studies. Qualitative analysis involved inductive content analysis of user features and thematic synthesis of HCP perspectives.

**Results:**

In total, 52 studies were included in the review, reporting on 44 professional-facing DHTs. The included DHTs were intended for remote patient monitoring, clinical decision support, assessment and diagnosis, education of HCPs, or a combination. The most common target population for DHT use was multidisciplinary care teams; the most common setting for implementation was primary care. Approximately half (26/44, 59%) of the professional-facing DHTs had a connected patient-facing system. Inductive content analysis of the user features produced 4 themes: guiding initial consultation, supporting chronic pain management, facilitating ongoing patient management, and supporting routine clinical duties. The thematic synthesis of HCP perspectives produced the following 4 themes, reflecting factors affecting the use of DHTs in chronic pain care: additional value, integration into clinical workflow, ease of navigation, and trust in the DHTs. Most (43/52, 83%) of the included studies did not adequately report appropriate stakeholder involvement in a proper co-design of DHTs; only 7% (3/44) of the DHTs were reported to have been developed with guidance from a system development framework.

**Conclusions:**

There are various DHTs available for HCPs to use in the management of chronic pain. The included studies neither reported adequate stakeholder involvement in the DHT development nor any specific frameworks to guide rigorous co-design. Therefore, future research should focus on developing professional-facing DHTs with active involvement of stakeholders in the design process.

**International Registered Report Identifier (IRRID):**

RR2-10.2196/51311

## Introduction

### Chronic Pain: Background

Chronic pain is a highly prevalent condition that is estimated to affect as many as 20% to 30% of individuals internationally [1,2]. Pain is distinguished as *chronic* when it lasts for ≥3 months [3]. Treatment for chronic pain can vary significantly, with approaches ranging from pharmacological interventions (eg, analgesics and opioid therapy) to nonpharmacological, alternative interventions (eg, cognitive behavioral therapy and physiotherapy) [4]. Chronic pain care and treatment can be particularly complex, as mismanagement can lead to significant adverse effects, such as opioid dependency [5]. Research contributing to the safe, effective treatment of patients with chronic pain is essential to reduce issues around mismanagement of chronic pain.

Due to its prevalence, pain is one of the main reasons why people seek health care [1]. This has resulted in a significant economic impact, with estimated costs of chronic pain to the economy reaching billions around the world [6-8]. Moreover, the COVID-19 pandemic has exacerbated these economic challenges, as health care resources were diverted from chronic pain care to more emergency situations [9]. Certainly, this combination of challenges has led to a reconsideration of traditional methods of care for chronic pain and has highlighted the importance of being flexible to more novel methods of health care delivery [10-12].

### Digital Health Technology for Chronic Pain

One of the ways in which chronic pain care can be addressed innovatively is through digital health technology (DHT). DHT can provide a unique opportunity to mitigate the challenges of chronic pain care in a cost-effective manner [9,13]. In this review, the term *digital health* will refer to all digital, electronic, and computer technologies to improve health, including eHealth, mobile health, telemedicine, or telehealth [14,15]. As definitions of digital health are continuously evolving and there is no universally accepted nomenclature, such terms are often used interchangeably in the literature [14,15]. Components of DHTs used for chronic pain may include mobile or tablet apps, web applications, wearable health technology, artificial intelligence, and telemedicine. Such DHTs can be used for the purpose of chronic pain self-management [16] or digitally delivered physical therapy [17]. DHT may be particularly beneficial for chronic pain care, as it addresses some of the aforementioned challenges by offering remote care and reducing the impact in certain areas of health care service provision, for example, lack of resources, waiting lists, and limited availability of the health care workforce [18,19].

### Health Care Professionals in Chronic Pain Care

Health care professionals (HCPs) are essential to effective chronic pain care; specifically, HCP involvement on a multidisciplinary level is integral to quality chronic pain care [2,20]. Furthermore, it has been suggested that chronic pain must go beyond simply *multidisciplinary* care and be targeted more holistically through *interdisciplinary* care, which denotes a more specific coordination of care and communication between HCPs from different disciplines [21]. Chronic pain is thought to have a biopsychosocial cause; therefore, care is best targeted through the coordination of the biological, psychological, and social factors contributing to the condition [22]. Moreover, primary care clinicians are thought to be especially important to effective chronic pain care [20,23]. Despite this, much of the literature on DHTs for chronic pain focuses on systems solely intended for patients (for self-management) without HCP involvement. HCP involvement has also been highlighted as an important factor and facilitator in the adoption of DHT, particularly as collaboration with key stakeholders in co-design is essential for the development of usable systems for sustainable implementation [24].

Previous studies on the perspectives of HCPs have also underlined the potential of DHT as a useful tool, for example, for HCP education and patient follow-up [19]. DHT, which targets HCP education, may have particular utility in improving chronic pain care, as negative attitudes and a lack of knowledge have been identified as significant barriers in chronic pain management [25]. In addition, national guidelines for chronic pain care state that HCPs must have the best possible resources and support to manage patients, which could potentially be supported by DHT designed for HCPs, that is, professional-facing systems [23].

### Previous Research

Previous reviews on DHTs for chronic pain have focused primarily on determining the effect on patient outcomes, with many of the investigated interventions being patient facing (ie, designed for the use of patients) for the purpose of self-management [18,26-29]. The results of these reviews show that DHT has positive outcomes for patients with chronic pain, such as reduced pain intensity, improved quality of life, coping skills, and adherence to exercise [18,26-29]. Although these reviews highlighted the utility of patient-facing DHTs to improve chronic pain management, it is equally important to focus research on and gain a more in-depth understanding of professional-facing DHTs. DHTs for HCPs in chronic pain care may help ensure evidence-based care and adherence to best practice guidelines, which would ultimately improve patient outcomes. Certainly, DHT for the use of HCPs in the management of chronic pain exists, for example, for clinical decision support (CDS) [30]. One previous scoping review investigated DHTs for musculoskeletal care available in the allied health industry but did not focus on chronic pain or nonallied health industry professionals [31]. However, to our knowledge, no previous reviews have investigated the available professional-facing DHTs for the care of patients with chronic pain.

### Review Question and Objectives

The question this scoping review will address is what professional-facing DHTs (ie, designed specifically for the use of HCPs) are available for the management of chronic pain?

Specific objectives include the following:

1. To investigate the components of existing professional-facing DHTs for the management of chronic pain, including (1) their target populations, (2) the settings they are implemented in (eg, hospital), (3) whether they are connected to a patient-facing DHT application, (4) what data they collect, (5) whether they are stand-alone or integrated into larger systems, and (6) their security and privacy considerations.
2. To investigate the methods with which the DHTs are designed and developed—whether they are developed with adequate stakeholder involvement in co-design and if they are guided by specific frameworks.
3. To analyze the user features of the DHTs.
4. To provide an overview of the outcomes of the DHTs measured by the included studies.
5. To analyze HCP perspectives on the use of DHTs in the management of chronic pain.

## Methods

### Overview

A systematic scoping review was conducted in accordance with the Joanna Briggs Institute (JBI) methodology for scoping reviews [32] and reported in accordance with the PRISMA-ScR (Preferred Reporting Items for Systematic Reviews and Meta-Analyses Extension for Scoping Reviews) statement for scoping reviews [33]. The completed PRISMA-ScR checklist is available in Multimedia Appendix 1.

### Protocol and Registration

The protocol for this scoping review was published in JMIR Research Protocols [34]. As this is a scoping review, PROSPERO registration did not apply.

### Eligibility Criteria

The inclusion of studies was guided by the population, concept, and context framework in accordance with the guidance in the JBI methodology for scoping reviews. A more detailed overview of the eligibility criteria following the population, concept, and context framework can be found in the study by McCartney et al [34].

#### Inclusion Criteria

Studies were included if they reported any DHTs designed and intended for the use of HCPs for the management of chronic pain among adults (Table 1). The types of eligible studies included interventional, observational, and descriptive studies fitting the inclusion criteria. No restrictions were placed on the time frame. The search was completed in July 2023.

**Table 1:** Description of the eligibility criteria of population, concept, and context (PCC) for study inclusion in this scoping review.

PCC	Eligibility criteria
Population	HCPs^a^ who were involved in chronic pain care (including but not limited to nurses, pharmacists, general practitioners, physiotherapists, occupational therapists, psychologists, and social care professionals) were eligible.
Concept	DHTs^b^ intended to assist HCPs in the management of adult (aged ≥18 y) patients with chronic pain (all types of chronic pain according to NICE^c^ guidelines and *ICD-11*^d^) were considered. DHTs must be designed to be chronic pain specific.
Context	There were no restrictions on context; research and clinical settings were considered.

^a^HCP: health care professional.

^b^DHT: digital health technology.

^c^NICE: National Institute for Health and Care Excellence.

^d^ICD-11: International Classification of Diseases, 11th Revision.

#### Exclusion Criteria

Studies were excluded if they reported DHTs that were not professional facing or chronic pain specific. Specific exclusion criteria are shown in Textbox 1.

**Description of the exclusion criteria.**

- Digital health technologies (DHTs) were considered not professional facing if the intended end users were not HCPs, that is, studies reporting self-management DHTs solely for patient use.
- DHTs were considered nonchronic pain specific if they were not specified as intended for chronic pain management or diagnosis (eg, DHTs for acute or general pain, nonspecific pain, or nonchronic cancer pain management). This also included studies reporting the use of technology on populations with chronic pain, which were designed for other uses (eg, general health care management and existing electronic health record or management systems not tailored specifically toward chronic pain care).
- Studies reporting nonadult care of chronic pain (aged <18 y) were excluded.
- Unpublished articles, protocols, and gray literature were excluded.
- Studies that were reported in a language other than English were excluded.

### Search Strategy

Comprehensive search strategies were developed using subject headings specific to the databases MEDLINE, Embase, CINAHL, PsycINFO, and Inspec (Multimedia Appendix 2). Search terms for chronic pain were developed using the terms used in the National Institute for Health and Care Excellence guidelines for the care of chronic pain among adults [35]. Further details regarding the development of search strategies can be found in the study by McCartney et al [34].

### Study and Source Evidence Selection

Search strategies were implemented in each database, duplicates were subsequently removed, and final search results were uploaded onto Covidence (Veritas Health Innovation Ltd). Two independent reviewers (HM and AM or MI) screened the titles and abstracts of the initial search results against the eligibility criteria. Following this, full texts of potential studies identified were screened again by 2 independent reviewers (HM and AM or MI). Authors of the studies that were unavailable were contacted to request access. Conflicts in both screening stages were resolved first through discussion and then through a third independent reviewer (HKR, NMW, or RM). Backward citation screening was also completed.

### Data Extraction and Quality Assessment

Data extraction and quality assessment of 20% (10/52) of the included studies were completed by 2 independent reviewers (HM and AM). As the rate of agreement between the 2 independent reviewers (HM and AM) for data extraction and quality assessment was high at 97% and 97%, respectively, 1 reviewer (HM) completed the rest of the data extraction and quality assessment work. The data extraction tool was adapted from the Template for Intervention Description and Replication (TIDieR) checklist [36], and the quality assessment was conducted using the Quality Assessment for Diverse Studies (QuADS) tool [37]. The adapted data extraction and quality assessment tool is available in the published protocol [34].

### Data Analysis and Synthesis of Results

Qualitative analysis methods were used to address review objectives 3 and 5. An inductive qualitative content analysis method was used to code the user features of DHTs reported in the included studies and group these codes into main category themes through a process of abstraction [38]. HCP perspectives, as reported by the included studies, were analyzed using the thematic synthesis method of extracting qualitative results, grouping codes into descriptive themes, and subsequently developing analytical themes, as reported by Thomas and Harden [39]. In accordance with this method, quotes presented in the Results section are direct quotes of study participants or key concepts and findings as reported by the studies. Qualitative analyses were conducted on NVivo (Lumivero) by HM and cross-checked by coauthors (AM, RM, HKR, and NMW).

## Results

### Study Selection

**Figure 1:**
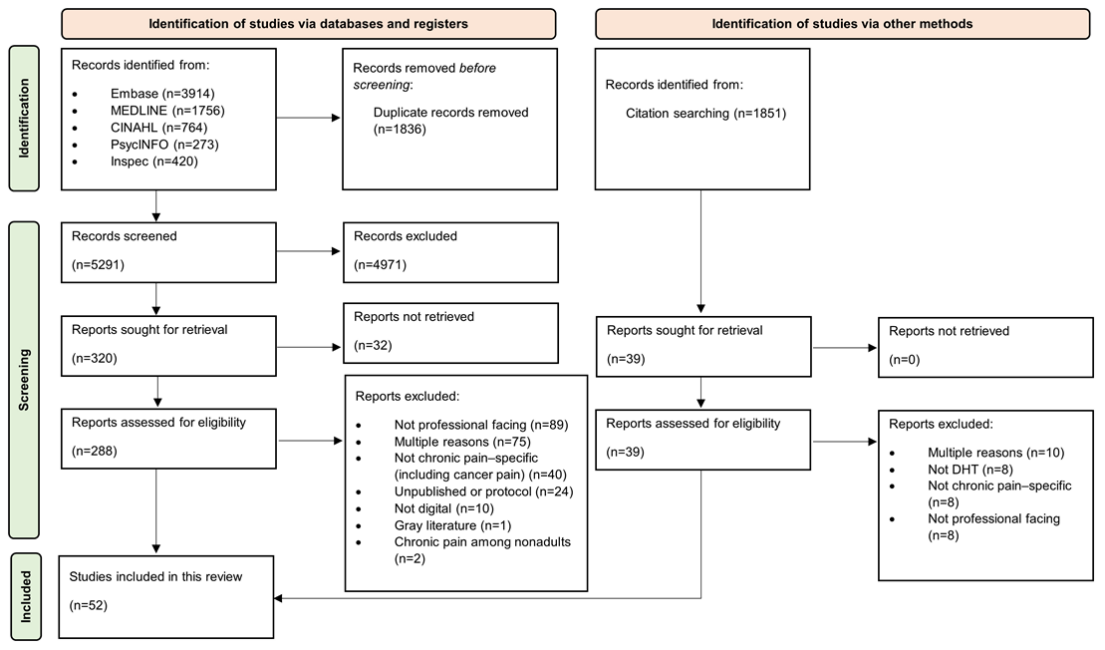
Flowchart to show selection of studies in this scoping review, adhering to PRISMA (Preferred Reporting Items for Systematic Reviews and Meta-Analyses) guidelines. DHT: digital health technology.

The initial search identified 7127 studies, of which 1836 (25.76%) were duplicates and subsequently removed. After screening full texts and backward citation screening, 52 studies reporting 44 DHTs were included. Reasons for exclusion are detailed in the PRISMA (Preferred Reporting Items for Systematic Reviews and Meta-Analyses) flowchart (Figure 1).

### Quality Assessment

QuADS [37] was applied to 46 (88%) of the 52 included studies. The remaining 6 (12%) studies were descriptive reports of the DHTs; therefore, QuADS could not be applied due to a lack of participant involvement and data collection [40-45] (as the QuADS criteria include description of target population, sampling approach, etc). The mean QuADS score was 22.7 out of 39 (SD 5.3), and the range was from 7 to 33. The criterion with the lowest quality score was “justification for analytic method.” Most (n=41, 89%) included studies did not provide a rationale for their chosen analyses, with the exception of 11% (5/46) studies [46-50]. Another criterion of lower quality was “evidence that the research stakeholders have been considered in research design or conduct,” with most (39/46, 85%) studies scoring 1 (out of 3) or below, apart from 15% (7/46) studies that described more substantial stakeholder collaboration in both the DHT development and planning of the research [49,51-56]. The criteria with the highest quality assessment scores were in reference to the appropriateness of study design and statement of research aims. The individual QuADS scores for the 46 included studies are available in Multimedia Appendix 3 [40-91].

### Review Objective 1: DHT Components

The 52 included studies of this scoping review were varied in their characteristics; further details regarding individual studies and DHT characteristics can be found in Multimedia Appendix 4 [40-91] and Multimedia Appendix 5 [40-91].

#### Target Population and Setting

The types of chronic pain that were targeted by the reported DHTs were as follows: general, unspecified chronic pain (12/44, 27%) [42-44,48,52,53,64-71]; chronic noncancer pain (5/44, 11%) [45,55,57-63]; a specific type of arthritis (including rheumatoid arthritis, osteoarthritis, spondylarthritis, inflammatory arthritis, and specific knee arthritis; 13/44, 30%) [40,46,47,50,54,72-80]; a combination of arthritis (2/44, 5%) [51,81]; chronic low back pain (5/44, 11%) [41,82-85]; chronic headache (2/44, 5%) [86,87]; spinal cord–related chronic pain (2/44, 5%) [49,56,88]; chronic musculoskeletal pain (1/44, 2%) [89]; endometriosis (1/44, 2%) [90]; and temporomandibular disorder (1/44, 2%) [91]. There were 16 different chronic pain conditions targeted by the DHTs. The most targeted chronic pain condition was arthritis, with 34% (15/44) of the DHTs specifically designed for arthritis care. The types of HCPs that were the target end users of the DHTs in each included study were also varied in their disciplines and were reported as follows: multidisciplinary teams (17/52, 33%) [44,46,48,49,53,54,56,66,68,72,75-80,82], a combination of primary care professionals (7/52, 13%) [45,55,63,64,67,71,89], primary care doctors (including GPs, internal medicine and family medicine; 5/52, 10%) [57,60-62,84], rheumatologists (3/52, 6%) [51,73,74], physiotherapists (2/52, 4%) [47,91], other therapists (1/52, 2%) [58], anesthesiologists (1/52, 2%) [70] and health coaches (1/52, 2%) [85]. Many studies did not specify the type of HCP and referred to their intended end user as “healthcare professional,” “clinician,” “clinical team,” or “healthcare provider” (8/52, 15%) [40,41,43,52,59,65,83,88].

The target settings for the DHTs also varied significantly across the included studies. The most common type of setting was primary care (12/52, 23%) [40,55,57,61-64,67,76,82,84,89]. Some of the included studies did target secondary and tertiary care settings, such as hospitals (5/52, 10%) [59,72,74,86,90] and specific chronic pain departments and clinics (11/52, 21%) [45,48,53,58,65,66,68-71,87] and rheumatology (6/52, 12%) [46,73,75,77,79,80]. Almost half (22/52, 42%) of the included studies reported implementation of the DHTs in the United States [40,41,43-45,48,50,53,55,57,60-64,66-68,71,80,85,88], with the next most common country of setting being the Netherlands (5/52, 10%) [75,79,82,89,91].

#### DHT Characteristics

In total, 18% (8/44) of the included DHTs were reported by multiple studies focusing on different stages of the development process, that is, initial requirement identification, user testing, etc [43-46,48,49,55-59,61-63,66,72]. The DHTs reported by the included studies fell into 5 distinct categories: DHTs aimed for remote patient monitoring (RPM; 22/44, 50%) [42,46-48,50-53,58,59,64-66,68,69,72,74-77,79-81,85,89], CDS (7/44, 16%) [40,45,60-63,71,73,82], assessment and diagnosis (2/44, 5%) [70,89], education of HCPs (3/44, 7%) [43,44,54,78] and a combination of the latter (eg, RPM and CDS; 10/44, 23%) [41,49,55-57,67,83,84,86-88,91]. RPM systems supported the HCPs in monitoring the patient’s chronic pain primarily by tracking their symptoms through patient-reported outcomes (PROs), treatment progress, or patient assessment completion. CDS systems supported the HCPs in making clinical decisions through an automated procedure of inputting data to produce an output (eg, treatment recommendation or suggested diagnosis), which was facilitated by a predetermined decision tree or algorithm. The most common type of professional-facing DHT was for RPM (25/44, 57%), including 7% (3/44) of the DHTs that had combined purposes of RPM and an additional area of care. Many of these DHTs also had a connected patient-facing DHT interface from which information regarding patient symptoms (PRO measure) was provided (26/44, 59%) [42,46-48,50-54,58,59,65,66,68-70,72,74-77,79-81,83,85,86,89,91]. The data collected by the DHTs were mostly patient-related data, such as results of clinical assessments (including PROs) on pain intensity, physical activity, disease activity, anxiety, or depression. Notably, 7% (3/44) of the DHTs collected patient data through a diary on a patient-facing interface [42,79,86]. Moreover, 2% (1/44) of the DHT solutions collected non–patient-related data—HCP user performance on an assessment (the purpose of which was education of HCPs) [78].

The included DHTs were designed to be accessed via different modalities; the most common modality was through online platforms and websites (19/44, 43%) [41-44,47,51-54,58,59,65,67,69,76,81-83,86,89,91], followed by computer applications or mobile and tablet apps (14/44, 32%) [40,45,46,48,50,55,57,61-63,66,70-72,79,80,87,88,90]. Notably, there was 1 virtual reality app that targeted the education of HCPs [78]. Many studies did not detail the specific modality of their DHT or reported use of an “interface” or “dashboard” (10/44, 23%) [49,56,60,64,68,73-75,77,84,85]. Most (34/44, 77%) of the DHTs were stand-alone systems (or did not report an ability to integrate); 22% (10/44) of the studies reported their DHT solutions as having the ability to integrate into larger systems, although they were still chronic pain specific [45,50,51,60-63,67,83,87]. Only 14% (6/44) of the studies specified the privacy and security considerations of the DHT solution reported, that is, the authentication method to log in or secure storage of patient data on the DHTs, although this was not always in great detail [46,51,52,65,79,89]. Further details of the included DHTs are provided in Multimedia Appendix 5.

### Review Objective 2: Methods of DHT Development

Many of the studies included in this scoping review did not focus specifically on the development of the DHTs. Less than half of the DHTs (20/44, 45%) were reported to have been developed with any stakeholder involvement, with most of the reporting studies (16/52, 31%) lacking specific details regarding the level of involvement. With regard to specific stakeholders involved in the DHT development, 34% (20/52) studies reporting 34% (15/44) DHTs described some HCP involvement [45,48,49,51,52,54-60,63,66,68,70,79,80,84,89]; 25% (13/52) of the studies reporting 25% (11/44) of the DHTs described some patient involvement [48,51,53,54,58,59,66,68,70,76,79,80,89]; 7% (3/52) of the studies reporting 7% (3/44) of the DHTs described some nonspecified “stakeholder” or user-tester involvement [40,60,76]; 9% (5/52) of the studies reporting 9% (4/44) of the DHTs described some other stakeholder involvement (eg, pain specialists or researchers, technical experts, and software engineers) [58,59,67,87,89]. Although stakeholder involvement in the development was briefly mentioned in these latter studies, few (9/52, 17%) studies reported any detailed or thorough co-design process. Notably, the development of only 11% (3/44) of the DHT solutions was reported to have been led by a system development framework [45,52,54,63], which was the analysis, design, development, implementation, and evaluation framework [92]; the Good Things Foundation pathfinder model for digital health inclusion [93]; and the Johns Hopkins University tool for development of clinical applications [94]. Only 4% (2/52) of these studies reported the use of a framework that focused specifically on digital health system development [52,54]. No studies reported the use of frameworks to guide both the intervention and system development. In total, 4% (2/52) of the DHTs were described to have been developed using the principles of user-centered [80] or decision-centered [55,57] design, but no specific frameworks were mentioned.

### Review Objective 3: DHT User Features

#### Overview

The inductive content analysis of the reported DHT user features gave rise to four themes that highlighted categories of HCP responsibilities in chronic pain management: (1) guiding initial consultation, (2) supporting chronic pain management (subtheme 2.1—managing opioid intervention and risk), (3) facilitating ongoing patient management (subtheme 3.1—managing PROs), and (4) supporting routine clinical duties. User features supporting the management of opioid treatment were separated into an individual subtheme within theme 2 of “supporting chronic pain management,” as consideration of opioid intervention is complex and is often considered separately in guidance for those with poorly managed chronic pain. Further details regarding individual user features can be found in Multimedia Appendix 5.

#### Theme 1: Guiding Initial Consultation

Many of the DHTs reported in the included studies involved user features that would help to support the HCPs in the initial consultation with the patient with chronic pain, which may include reviewing available patient information and diagnosis. This included functions that would assist the HCP in familiarizing themselves with the patient’s clinical background, such as access to patient medical history (current and past treatments) and past assessment results. Access to patient clinical background and history through the included DHTs was the most reported user feature overall, with 34% (15/44) of the DHTs having this function available to HCPs [40,45,48,49,52,56,60,63,66,69,73-76,79,81,84,90]. The initial consultation may also include initial assessments, such as consideration of “red flags” and identification of risks (eg, medical history indicating inclination to opioid dependency), which was supported by user features, such as access to risk assessments. Diagnosis of chronic pain was supported by user features of many included DHTs, including access to clinical assessments (such as the Brief Pain Inventory [95,96] and the Pain Catastrophizing Scale [97]) and, most notably, through diagnostic decision support (ie, using predetermined algorithms to generate automatic diagnosis; 8/44, 18%) [41,49,56,70,84,86-88,90]. A total of 58% (30/52) of the included studies reporting 57% (25/44) of the DHTs included user features that guided the HCP in the tasks involved in initial assessment; most of these DHT solutions were designed for RPM (12/44, 40%).

#### Theme 2: Supporting Chronic Pain Management

##### Overview

The second categorical theme highlighted the user features of the DHTs, which would assist the HCPs following the initial consultation of the patient, which may be the stage in which a practitioner would make decisions regarding treatment for pain management. The supported functions of the DHTs that facilitated this included the generation of future treatment recommendations via CDS, the ability to prescribe medication (directly through the system), and direct access to treatment programs (ie, to physiotherapy materials). The ability of the HCPs to generate treatment recommendations using DHTs was the most commonly reported user feature supporting chronic pain management (9/44, 20%) [48,55,57,60-62,64-66]. In addition, 37% (19/52) of the included studies reported 34% (15/44) of the DHTs with user features that facilitated HCP decisions regarding intervention, with the most common type of DHT being for CDS (9/44, 20%).

##### Subtheme: Managing Opioid Intervention and Risk

A subtheme within this category of DHT user features supporting HCP management of chronic pain was “managing opioid intervention and risk.” This subtheme categorized the DHT user features that helped the HCPs specifically regarding opioid intervention for chronic pain. Functions supported by the reported DHT solutions that fell into this categorical theme included visualization of both the patient’s current and previous opioid interventions [48,57,60-62,64-66] and access to patient opioid agreement or consent documentation [63,64]. Moreover, 21% (11/52) of the included studies reporting 16% (7/44) of the DHTs addressed the task of managing opioid intervention and risk; there was an even split between the type of DHT with this category of user features, with 7% (3/44) of the DHTs promoting CDS, 7% (3/44) of the DHTs aiding RPM, and 2% (1/44) of the DHTs supporting a combination of CDS and RPM.

#### Theme 3: Facilitating Ongoing Patient Management

##### Overview

This theme described the user features that supported the HCPs in facilitating ongoing management for patients, which would follow initial consultation and prescribing of intervention. This was the task most commonly supported by the DHT user features, for example, to review treatment effectiveness or prepare for follow-up consultations (ie, reviewing information inputted by patients before the consultation). Included studies reported on DHTs that provided the HCPs with information (ie, assessment results) on patient pain, physical activity, psychosocial and anxiety symptoms, sleep, disease activity, and patient goals, which could be used to continuously review patients (8/52, 18%) [40,41,49,56,60-62,67,70,73]. The most common type of DHT with user features to assist the HCPs for ongoing patient management was RPM (25/44, 57%), presented by 77% (40/52) of the studies.

##### Subtheme: Managing PROs

Many of the DHTs with user features to support ongoing management reported having the ability to display patient-reported symptoms from connected patient-facing DHTs, including pain levels, physical activity, disease activity, quality of life, psychosocial symptoms and anxiety, sleep, and physical symptoms (heart rate and blood pressure; 26/44, 59%). These connected patient-facing DHTs were mostly intended for the patient to self-manage their chronic pain. Some (17/44, 39%) of the DHTs were described to display patient symptoms in a graphical format or a body map, and other (8/44, 18%) DHTs allowed HCPs to have direct access to a patient diary or log from a connected self-management DHT and assign specific PRO measures for patients to complete if they required further information for review. Moreover, 20% (9/44) of the DHTs allowed HCPs to communicate with patients using a chat function that was connected to the patient-facing interface, eg, to allow patients to ask HCPs questions regarding the intervention [46,72] and have general communication with the care team [76,89] or for HCPs to give feedback to patients regarding care [47]. Notably, some (4/44, 9%) of the DHTs included features that allowed HCPs to be alerted to situations that required more immediate attention, for example, due to worsening disease activity [51,52,74,89].

#### Theme 4: Supporting Routine HCP Duties

This final categorical theme highlighted user features that supported other duties of the professionals that may be viewed as more routine, that is, tasks that required attention more frequently, and not necessarily within consultation with patients, by all HCPs, regardless of discipline. This includes user features of the DHTs that allowed HCPs to carry out general health care–related duties, such as functions for refreshing clinical knowledge to provide evidence-based care (accessing chronic pain–specific educational materials, such as direct resource access links, case scenario assessments, and up-to-date chronic pain guidelines) and for more operational duties (access to individual HCP caseloads, the ability to see and modify clinical notes and patient appointments, and notifications for new tasks to complete). The most common function within this category was to support the HCPs in refreshing their knowledge of chronic pain, including access to care guidelines (11/44, 25%) [43,44,54,55,57,60-62,67,71,78,79,82,90]. Moreover, 2% (1/44) of the DHTs also allowed HCPs to contact other professionals [76]. User features of this category were possessed by 39% (17/44) of the different DHTs reported by 40% (21/52) of the studies, with the most common type of DHT being for RPM (8/44, 18%).

### Review Objective 4: Overview of the Outcomes

Due to the significant heterogeneity of aims and design of the included studies, there was also significant variation in the outcomes measured. Less than half (24/52, 46%) of the included studies focused on outcomes related to the HCPs (eg, HCP perspectives on the DHTs and rate of DHT use) [44,50,53-55,57,60-64,66,67,69-71,73,78-80,82,84,89,91]. In addition, 2% (1/52) of the studies reported measuring HCP-related outcomes on DHT user experience but did not specifically report the results [52]. There were some studies that focused solely on patient outcomes (eg, clinical patient characteristics of change in pain intensity, quality of life, and physical activity or intervention efficacy and adherence or patient perspectives on the DHT; 14/52, 27%) [51,56,58,59,65,68,72,74,75,77,81,85,86,90]. Cost-effectiveness of the DHT implementation was also measured as an outcome in 6% (3/52) of the studies included in this review [46,47,76].

### Review Objective 5: HCP Perspectives on the Use of DHTs in Chronic Pain Care

#### Overview

**Figure 2:**
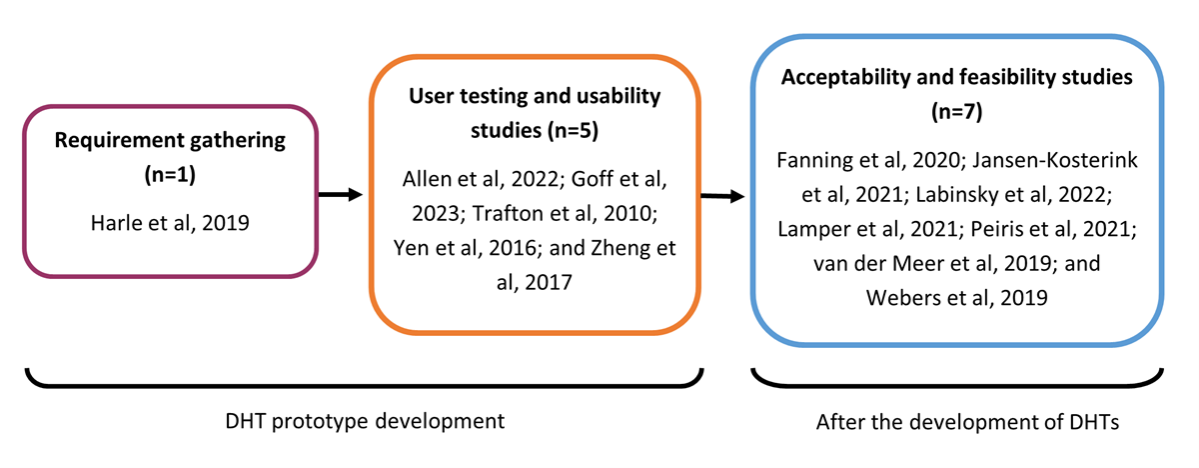
Qualitative studies included in this scoping review reporting health care professionals’ perspectives on digital health technology (DHT) for chronic pain management, divided into stages of DHT development (n=13) [51,54-56,58,64,74,80,81,83,85,90,92].

Some (13/52, 25%) studies reported HCP perspectives through qualitative methods, such as interviews and open-response questionnaires. Studies ranged in their aims, falling into two categories of DHT “development” and “after development”: (1) some (6/52, 12%) studies investigated HCP perspectives for developing and refining a DHT prototype (user testing or usability studies) [50,54,55,57,63,80] and (2) some (7/52, 13%) studies investigated HCP perspectives on their experience in using a developed DHT system (acceptability and feasibility studies) [53,73,79,82,84,89,91] (Figure 2 [50,53-55,57,63,73,79,80,82,84,89,91]). Many of these qualitative studies defined assessment of acceptability and feasibility in different ways; for this scoping review, acceptability and feasibility refer to the included studies that focused on qualitatively investigating HCP perspectives following the development of a DHT prototype.

The thematic synthesis of these studies gave rise to four analytical themes describing the factors that affected HCP use of DHTs in chronic pain care: (1) additional value of DHTs (with 3 subthemes), (2) integration of DHTs into clinical workflow (with 2 subthemes), (3) ease of DHT navigation, and (4) trust in DHTs.

#### Theme 1: Additional Value of DHTs

HCPs expressed that the DHTs must add value by improving their professional experience beyond what was already offered by working nondigitally. The factors that contributed to the value of the DHTs were whether the DHTs helped facilitate HCPs’ clinical duties, whether the DHTs improved visualization of comprehensive patient information, and whether the DHTs consequently enhanced patient care and outcomes.

##### Subtheme 1.1: Facilitating HCPs’ Clinical Duties

A highly reported experience of the HCPs was the desire for the DHTs to facilitate clinical duties in a way that was more efficient. DHTs were seen as useful for a variety of clinical duties; for example, the DHT was observed as being useful in “aggregating critical information that, in their current practice, was often scattered...and took time to find and synthesize into a coherent whole” [57]. The DHTs were helpful in “work[ing] consistently with evidence-based medicine...support during decision making” [82] and providing “additional information” [81]. Some viewed the DHT as “an extra treatment option above the existing option” [89]. User testing studies also highlighted HCP suggestions for adding value to DHT usefulness in clinical duties, for example, “adding arthritis guidelines and setting it as a goal line in activity graphs” [80]. Some HCPs saw the additional value of the DHTs in improving their clinical practice, depending on the context, that is, “positive experiences with [the DHT]...was mostly in a blended form” [91]. Barriers to DHT implementation were also expressed by some HCPs, with some seeing the DHTs as not adding value to their clinical duties, for example, an HCP noted that “you can’t have a tool for every presentation in general practice...this is something that should be up there [pointing to head] rather than ‘oh wait a second, let me use this tool’” [84].

##### Subtheme 1.2: Visualizing Comprehensive Patient Information

One of the main reported ways in which the DHTs had additional value was in improving the visualization of comprehensive patient information for the HCPs. HCPs felt a “strong need...to be presented with comprehensive patient history” in the development of the DHT [57] and found the DHT to be useful for their clinical practice when this was provided in various ways; for example, authors reported, “[HCPs] liked summary data in a trend format that ‘gives a nice, quick snapshot’ [and this is] ‘helpful’ for [the HCP] and patient to discuss issues and change treatment” [80]. Included studies described that “care providers appreciated the additional information for [preparing] their consultations, the insight gained from the evolution of important outcomes...over time in relation to medication use” [79]. Some HCPs thought that “data visualization is in general much better with [the] tool” [73] and that DHT’s “graphical display of the results was especially of added value as it gave insight into the efficacy of the treatment” [89]. HCPs also suggested ways in which the DHT could better the visualization of patient information; that is, authors reported that the HCP “thought that while daily reports of pain or symptoms would be good for patient’s monitoring, [surgeons] would like to see summary changes by week or by month to make decisions” [80].

##### Subtheme 1.3: Improving Patient Care and Outcomes

The included studies reported that a large contributor to HCPs’ experience of DHT value was ultimately the impact on patient care and outcomes. HCP perspectives highlighted the usefulness of the DHT in improving patient care; for example, “I think patients get the most annoyed when I suggest something that we have done before...so I think patients would like the most that I’ve remembered (in reference to having access to patient history through DHT)” [57]. HCPs expressed that the reported DHT “could be beneficial to many patients” [50]. Specifically, the included studies suggested the added value of the DHT for patient care in implementing “shared decision-making” [73], “patient reassurance” [84], and “personalized [patient] care...increasing self-efficacy [for the patient]” [91], “patient empowerment” [82], and “better streamlining of care [leading to] better treatment results for the client” [82]. Much of this referred to DHTs, which had a connected network of professional-facing and patient-facing systems. However, barriers to DHT use were highlighted as hindering patient care and outcomes by some HCPs, particularly that the DHT could be at the “expense of patient-clinician interaction” [79] and “personal contact” [91].

#### Theme 2: Integration of DHT Into Clinical Workflow

A major factor in the implementation of DHTs was the ability of the system to adapt to the HCPs’ clinical workflow. HCPs expressed a desire for the DHTs to easily accommodate their current clinical environment and for there to be a balance in the time, effort, and cost of implementation into clinical practice.

##### Subtheme 2.1: Adapt to Current Clinical Environment

HCP perspectives emphasized the importance of the adaptability of the DHT into their current clinical environment; that is, they would not have to significantly alter their responsibilities to accommodate the use of the DHT in question. This was mostly reported by the HCPs as a source of challenge, with few commenting on the utility of the DHT in adapting to their current clinical environment. HCPs expressed that the issues could arise in adapting to the physical environment, for example, “a lack of networked printers limited the usefulness of the [DHT]...software or hardware changes led to slowing of the system to the point that it was...disruptive to workflow” [63]. There was also reference to more logistical concerns; for example, authors described HCP concerns over “the implementation of such a mobile application into the clinical workflow and how it may impact patient care” [50] and that some HCPs “indicated that it is difficult for them to combine the [DHT] with other existing applications in their daily practice” [89], suggesting better integration into the professional routine (eg, “the main influence to future tool uptake appeared to be integration into routine workflow” [84]).

##### Subtheme 2.2: Balance in Time, Effort, and Cost

In line with HCPs’ concerns regarding DHT adaptability to their current clinical environment, HCP perspectives underlined balance in time, effort, and cost as a factor in their use of DHTs in chronic pain care (due to the lack of time in HCP work schedules). HCPs reported that, for successful integration of the DHT into the clinical workflow, implementation must neither significantly increase their time and effort nor the financial cost. DHTs were seen as having a positive influence on the amount of HCP time and effort in clinical practice, for example, in “workload reduction, as the [DHT] could also be used by the practice nurse” [82]. Authors stated that “GPs indicated that the patient self-registration route was timesaving for them” [89]. A balance in these factors is essential, as HCPs expressed the need for time efficiency and “brevity” of information (ie, “quick scanning” [63]) while also desiring comprehensive patient information (as stated in subtheme 1.2). HCPs were skeptical about “having adequate time to use the [DHT], given time-constrained work schedules” [57] and “saw an extra investment in time, money and administration” [91]. In terms of financial cost, HCPs found it “concerning” as costs were likely to increase with expanding future use [89]. Balance was also key regarding financial cost, as some HCPs were reported to perceive that “some [of the DHT] features were worth this investment” due to its additional value [91].

#### Theme 3: Ease of DHT Navigation

A frequently mentioned factor in DHT usefulness by the HCPs was ease of navigation, regarding its user-friendliness and accessibility. User testing studies in the development of the DHT prototype brought attention to HCPs’ system-specific suggestions to increase usability (eg, to address “confusing” navigation [50]) and accessibility (eg, changing data entry modules for increased accessibility for HCPs with color blindness [50]). Some HCPs praised the DHT for its ease of navigation; for example, a study reported that “overall, [HCPs] indicated that the [DHT] is easy to use” [89], and some practitioners thought that the DHT in question was “user-friendly” [82,91]. Most comments regarding issues in DHT navigation were reported in studies addressing prototype development and were subject to improvement in the future; few after development studies identified any barriers in reference to navigation.

#### Theme 4: Trust in the DHT

HCPs conveyed a need to trust the DHT in providing good care, that is, relying on the clinical accuracy of the information provided as well as the quality of the system. Some HCPs highlighted some concerns about the DHT containing “accurate, trustworthy, and relevant information” [57]. Practitioners expressed that “one of the challenges is who put the information there?...the information has to be verified” [57]. Accuracy issues with the DHT “can generate insecurity in the user” [73]. Studies on user testing highlight HCP suggestions to improve the clinical accuracy of information provided by the DHT; for example, authors reported that there was “helpful feedback on the wording of [graphical user interface] elements, particularly the level of detail and vocabulary level most appropriate for primary care providers” [63]. Some HCPs also experienced concerns about the quality of the DHT (eg, “underlying electronic health record data quality and...the ability...to consistently display the most relevant information” [57]), while other HCPs chose to use their system due to its high quality (eg, a study reported “the six [orofacial physical therapists] who started using [the DHT] during the COVID-19 pandemic chose this platform due to its available content and its quality, and the positive experiences of other colleagues in the field” [91]). Regardless of this conflict between HCP opinions on specific DHTs, it is clear that both the quality of the intervention content and the DHT system are important factors in HCPs trusting the DHT and consequently its implementation.

## Discussion

### Principal Findings

To our knowledge, this is the first scoping review to systematically investigate professional-facing DHTs for the care of patients with chronic pain. This scoping review identified that various professional-facing DHTs to support chronic pain management exist, with 52 of the included studies reporting 44 of the DHTs aimed at RPM, CDS, assessment and diagnosis, and HCP education (or a combination).

The first objective was to investigate the components of professional-facing DHTs, which varied significantly. The most commonly targeted population and setting of DHT use were multidisciplinary teams of HCPs for the care of arthritis (various types) in primary care. Over half (26/44, 59%) of the reported DHTs have a connected patient-facing interface, mostly for the purpose of RPM through patient-reported data collected on self-management mobile apps. The second objective was to investigate the methods of DHT development; many of the included studies did not specify this. Less than half (20/44, 45%) of the DHTs were reported to have been developed with any form of stakeholder involvement, and fewer (7/44, 16%) DHTs were reported to have been developed with enough stakeholder involvement to constitute proper co-design. The third objective was to analyze the user features of the available professional-facing DHTs. The user features support the HCPs in duties to guide initial consultation with patients, support chronic pain management for patients (including management of opioid intervention and risk), facilitate ongoing patient management following initial consultation (including management of PROs), and support more routine tasks, that is, daily tasks that can occur outside of patient-professional interaction. The fourth objective was to provide an overview of the outcomes of the studies reporting the available professional-facing DHTs, which differed significantly across the included studies. Notably, less than half (24/52, 46%) of the included studies reported specific, professional-related outcomes, and some (14/52, 27%) focused solely on PROs. Finally, HCP perspectives suggested that the DHTs must fulfill the requirements mentioned subsequently for successful implementation in practice. The DHTs must have additional value to the HCP, must easily integrate into the clinical workflow of the user, and must be easy to navigate, and HCPs must be able to trust the DHTs.

### HCPs and Digital Health

The findings of this review are in line with previous literature that focuses on digital health for professionals in health care. DHT has many benefits for HCPs, as it reduces clinical workload while enhancing work efficiency, therefore providing both financial and operational advantages [98]. Previous literature has highlighted DHT as a solution to mitigate some of the issues surrounding chronic pain management, such as limited resources for care [18,19,23]. This has been of particular value in the post–COVID-19 pandemic world, where health care services have been strained [98]. Digital health provides unique opportunities for HCPs, as a large proportion of the world’s population has access to the internet and smart technology, opening new possibilities for remote monitoring of patients [99]. Remote monitoring may be one of the main ways that DHT can provide benefits to both the HCP and patient, as it could improve professional efficiency [98], which consequently would have an impact on patient outcomes. The potential of remote monitoring through DHTs is clearly a focus for exploration in digital health research on chronic pain. This is supported by the findings of this review in several ways. First, many (22/52, 42%) of the reported DHTs were designed primarily with the aim of RPM. Second, most (26/44, 59%) of the DHTs had a connected patient-facing interface that could be used to monitor patient progress. Third, the main outcomes measured by the DHTs were PROs. Fourth, the analyses of the DHTs resulted in a category of user features that can aid the HCP in facilitating ongoing patient management, particularly through management of PROs. Fifth, the synthesis of HCP perspectives showed greater visualization of comprehensive patient information as a factor that would encourage professionals’ use of DHTs, which would be most significant in the remote monitoring of patients.

Indeed, it is important to take into consideration the needs of the HCPs when considering chronic pain care [100]. Professional acceptance of DHT has been suggested to be influenced by the amount of time or work required to use the system and the relevance of system information [101], which aligns with the results of this review. This review highlighted that HCPs place importance on the ability of the DHTs to easily integrate into the clinical workflow and the trustworthiness of the DHTs, which is in concurrence with the reasons for professional acceptance as reported by previous literature [101]. For the most part, HCP perspectives were similar across the included studies with regard to the factors affecting their use of DHTs, despite the significant heterogeneity in the types of DHTs they were considering. This general agreement suggests that these factors should be taken into consideration when designing any DHTs for HCPs. Such factors that affect professional acceptance of DHT use in chronic pain care are reflected in the literature focusing on other areas of digital health care; previous research on other conditions, such as chronic obstructive pulmonary disease and hypertension, has highlighted facilitators of HCP use of DHTs, including ease of integration into the clinical workflow [102], high usability [102,103], and the additional value of the solution (for greater access to patient data and to increase patient engagement and communication) [102,104,105]. However, despite research being conducted on HCP perspectives on digital health, professional-facing DHTs may not be meeting their full potential of benefits due to a lack of HCP acceptance and design input [106]. This may indeed be the case for the available DHTs identified by this review, as the level of HCP input into the design was inconsistent across the literature.

The idea that there is a further, unmet potential of DHT that is directed at HCPs to support chronic pain management is reinforced by several findings of this review. Although this review testifies not only to the availability but also to the variety of professional-facing DHTs, few DHTs that were identified would improve the provision of holistic and interdisciplinary care. In fact, only 1 of the DHT solutions supported a feature that would allow HCPs to communicate with one another to coordinate care [76], despite the main target HCP population for DHT use being multidisciplinary care teams. As mentioned earlier, digital health is explored as a method to complement traditional care as it introduces the potential opportunity to mitigate some of the challenges surrounding chronic pain care, such as a lack of resources [18,19]. The literature on chronic pain care emphasizes the importance of holistic care, particularly as the evidence from research suggests biopsychosocial mechanisms of chronic pain [22]. The lack of resources is a major reason why many individuals with chronic pain are unable to access the specialized care that would promote evidence-based, interdisciplinary, and holistic management, which is seen as the most ideal standard of chronic pain care [21,23]. DHT may provide a solution to this issue and be one way to make interdisciplinary care more accessible in the future. Certainly, DHT for professionals in health care is important to support evidence-based practice for HCPs and subsequently improve patient outcomes.

### The Importance of Co-Design

It is accepted that the involvement of stakeholders, particularly HCPs and patients, in the design of DHTs is important for ensuring usability and successful implementation in health care practice [107-109]. Despite the recent rise in digital innovations for chronic pain [52] and the abundant availability of professional-facing DHTs as demonstrated by this review, much of the research supporting DHT development still lacks adequate reporting of appropriate stakeholder involvement. Over half (24/44, 54%) of the DHTs included in this review were developed without any form of collaboration with stakeholders. Furthermore, the quality assessment of the included studies identified involvement of stakeholders as a criterion of low quality. All of this suggests that the literature in this research area is still suboptimal, as it lacks consistency in the reporting of digital health development. For standardization in the reporting of digital health, the CONSORT-EHEALTH (Consolidated Standards of Reporting Trials of Electronic and Mobile Health Applications and Online Telehealth) checklist [110] was created and is occasionally consulted by clinical trials of DHTs; a criterion within this checklist is “describe the history/development process.” It is clearly important to be able to access information regarding the specific development process of DHTs. This is a criterion that many of the studies included in this review would fail to meet. This may also be due to the CONSORT-EHEALTH checklist being a guide for randomized controlled trials in DHT research, which most (47/52, 90%) of the included studies are not. To our knowledge, no guidelines exist for the standardization in the reporting of studies to develop DHTs, which mostly use qualitative methods. This may be a major reason for inconsistencies in the literature on the reporting of DHT development.

Although necessary due to its complexity, digital health in general often lacks stakeholder involvement, consequently limiting its adoption [107]. Again, this may be due to a lack of guidance on how to adequately use co-design in digital health research [107]. Co-design refers to a method essential in DHT design, which involves direct and active involvement of stakeholders (most importantly, the end users) in the whole design process from the early stages of conception [107-109]. This method is not consistently described or evaluated in the literature [111], which is reflected by the studies included in this review. For the most part, the studies that did describe some level of stakeholder engagement in the DHT development did not consistently report methods that met the co-design criteria of active collaboration across the whole process from design planning and conception. Although this suggests that these studies did not practice proper co-design in their DHT development, it may also be that the studies did not adequately report the involvement of stakeholders. The challenge of properly applying co-design methods in digital health research is thought to be due to high HCP workload, resulting in a lack of time for sufficient active involvement [108]. However, increased time and effort for co-design application could lead to adoptable DHTs, which can eventually improve work efficiency for professionals [98], which in turn will inevitably have an impact on both professional and patient outcomes.

### Implications for Future Research

The results of this review show good availability of professional-facing DHTs. However, the methods of DHT development are still lacking due to several reasons. First, it is unclear from the included studies whether any of the DHTs were developed through methods that adequately meet the standards of proper co-design; second, there is no evidence to suggest that any of these studies followed specific frameworks to guide both the intervention development and the system development. The use of frameworks would ensure that the design methods are rigorous to result in a DHT that is evidence based, with reliable clinical information and high usability. Both are equally important to consider for DHTs and should adhere to the principles of co-design through adequate involvement of end users. HCP perspectives of this review also strengthen this argument, as the ability for the professionals to trust the DHTs to provide clinically accurate information and usability were highlighted as factors that would encourage the use of digital health for chronic pain care. Focusing on the rigorous development of the intervention and the background system through the use of frameworks would result in the fulfillment of the latter factors. For intervention development, frameworks exist, such as the Medical Research Council framework, which highlights the importance of iterative and rigorous development through an evidence-based approach of research and stakeholder engagement [112,113]. Iterative development has also been highlighted as particularly useful for improving the usability of DHTs [114]. Models that focus specifically on user-centered principles of DHT development, such as the Centre for eHealth Research Roadmap, also exist to guide system development [115]. These types of system development frameworks are beneficial to DHT research, as they focus more on usability. Future research on the development of professional-facing DHT should use rigorous co-design through active involvement of stakeholders from the beginning of the process and may also benefit from using intervention- and system-focused frameworks, which encourage co-design principles. This will help in ensuring evidence-based assistance for professionals, increased usability, and consequently, more sustainable adoption of DHTs.

In general, future research should also focus more on how DHTs can help HCPs in providing chronic pain care. There is a clear interest in the potential for DHTs to improve chronic pain care from the professional perspective, as demonstrated by the rationale supporting the studies included in this review. However, much of the literature still focuses more on the patient perspective; most (28/52, 53%) of the studies included in this review did not measure professional outcomes, focusing more on the connected patient-facing DHTs. Future research on professional-facing DHTs should also ensure to measure professional outcomes and not just patient outcomes, for example, HCPs’ use of DHTs. Moreover, there are many literature reviews that focus on patient-facing DHTs, but few exist on professional-facing DHTs for chronic pain management. This scoping review was the natural first step to conducting further research on this topic. To our knowledge, there are no systematic reviews that investigate the effectiveness of professional-facing DHTs for improving chronic pain care. As per the guidance in the *JBI Manual for Evidence Synthesi*s [31], a scoping review, which focuses on intervention availability, forms the previous step to a systematic review, which focuses on intervention efficacy. A systematic review may be necessary in the future to fully investigate the effectiveness of DHTs for HCPs in chronic pain health care.

### Implications for Future Development of DHT for Practice

As discussed, the available DHTs for professionals in chronic pain care are heterogeneous but may not necessarily meet the full potential for aiding HCPs due to a lack of professional acceptance and design input [106]. In addressing the objectives of this scoping review to investigate the professional-facing DHT components, user features, and HCP perspectives, several gaps were identified that could be addressed to improve chronic pain care practice through digital health.

The lack of focus on providing holistic chronic pain care suggests that there is a potential gap for a professional-facing DHT solution that supports interdisciplinary care of chronic pain, allowing greater coordination of care and communication between multidisciplinary HCPs. The most commonly targeted setting for DHT implementation in this review was primary, which is where most chronic pain conditions are cared for in the United Kingdom [23,35,116] and around the world [20]. Perhaps the impact of chronic pain is greatest on primary care, especially economically, which is a significant burden [116]. There may be an unmet potential in DHTs to support primary care more comprehensively or improve interdisciplinary care of chronic pain. However, context is also important to consider for future developers, as the areas of care in which chronic pain has the greatest burden may be variable depending on the health care system of the country.

Despite professional acceptance being essential, some of the system components of the reported DHTs conflict with the themes highlighting HCP perspectives. Most (34/52, 65%) of the included studies reported their DHTs to be standalone or did not mention an ability to integrate into larger, existing systems; however, the thematic synthesis highlighted integration into the current clinical workflow of the professional as a factor that would encourage the use of DHTs. Indeed, some DHTs may have this ability to integrate even if the included studies did not report this. The lack of the ability to integrate, exhibited by many of the existing DHTs, is actually emphasized as a factor that inhibits wider clinical use in studies included in this review due to inconvenience to the HCP [87]. For optimal application of professional-facing DHTs in practice, future DHTs may be best developed with the ability to integrate into existing systems to ensure easier assimilation into the clinical workflow of the end user professional. Similarly, despite the trustworthiness of DHTs being valued by HCPs, few (6/52, 12%) studies reported any details regarding security and privacy. This is extremely important for the adoption of any professional-facing DHT solutions in practice because such systems will likely contain sensitive patient data that require protection. Developers of such systems should consider the factors inferred from the thematic synthesis of HCP perspectives on the use of DHTs for chronic pain management, ensuring that the system has additional value, integrates into the clinical workflow, is easy to navigate, and promotes professional trust in providing quality care.

### Strengths and Limitations

This is the first scoping review to investigate professional-facing DHTs for the care of patients with chronic pain. It is also the first review to conduct an analysis of the user features that support HCPs in chronic pain care through digital health as well as the first to thematically synthesize HCP perspectives on the use of DHTs specifically designed for professionals. Therefore, it is likely that this review is the most comprehensive report of chronic pain–specific DHTs that support professionals in health care. A major strength of this scoping review is the systematic process used to screen reports for inclusion. A comprehensive search strategy was cross-checked by an academic librarian, and results were screened by 2 independent reviewers for study inclusion. Furthermore, this review followed the scoping review guidelines set by the JBI and PRISMA to ensure rigor. Therefore, it is likely that most of the literature that supports professional-facing DHTs for the care of patients with chronic pain was captured by this review.

However, several limitations of this review exist, which may have resulted in some relevant articles being missed for inclusion. First, the search was limited to studies written in the English language; therefore, systems from non–English-speaking countries may have been missed. This is particularly important to note as a limitation, because there was significant variability in the countries the available DHTs were developed and designed for application in the health care systems of; other studies that report on updates or adaptations of the included DHTs may be reported in non-English languages. In addition, the search was limited to academic databases and excluded gray literature, and DHT solutions may be developed commercially or without publication of peer-reviewed research. Nonetheless, the interest of this scoping review was the availability of interventions that are evidence based and therefore more likely to be implemented in real health care settings. This review showed that many of the studies reporting professional-facing DHTs focus primarily on the connected patient-facing DHTs and their impact on patient outcomes, with little information regarding the professional interface. Thus, there is a possibility that other relevant studies may have been excluded during the screening process due to a lack of focus on the HCP and resultant absence of information on the professional-facing DHTs in the abstract. Finally, some relevant studies may have been missed due to the nomenclature of chronic pain. There is some variability in the chronic pain literature on how the condition is defined, that is, how long pain must be experienced before “acute pain” turns to “chronic pain” [117]. This review used the *International Classification of Diseases, 11th Revision* guidelines of pain experienced for ≥3 months, or if reported by the authors specifically as “chronic,” to assess eligibility. Some relevant studies may have been missed in the search if any other definition of chronic pain was used or if they did not specifically report that the DHT was for chronic pain care.

### Conclusions

This scoping review is the first to investigate the availability of professional-facing DHTs in the care of patients with chronic pain. There is a clear interest in DHTs for chronic pain care for use by HCPs, as highlighted by the availability and variability of such solutions. However, the focus on professionals in this research area is still limited. There is still some unmet potential in professional-facing DHTs. The interest in digital health research, specifically on remote monitoring of patients, shows an unprecedented opportunity for greater access to and ease of care for both the patient and professional, which can be explored further in future research on DHTs for chronic pain care. Research reporting on professional-facing DHTs is lacking in proper co-design development. Future developers of professional-facing DHTs should consider this when designing a new intervention.

## Data Availability

All data generated or analyzed during this study are included in this published article (and its supplementary information files).

## Appendix

Multimedia Appendix 1 PRISMA-ScR (Preferred Reporting Items for Systematic Reviews and Meta-Analyses Extension for Scoping Reviews) checklist.

Multimedia Appendix 2 Search strategies.

Multimedia Appendix 3 Quality Assessment for Diverse Studies scores for included studies.

Multimedia Appendix 4 Characteristics of studies included in the scoping review (N=52).

Multimedia Appendix 5 Intervention characteristics (including the most common user features reported, divided by category; n=44).

## Abbreviations

CDS: clinical decision support
CONSORT-EHEALTH: Consolidated Standards of Reporting Trials of Electronic and Mobile Health Applications and Online Telehealth
DHT: digital health technology
HCP: health care professional
JBI: Joanna Briggs Institute
PRISMA: Preferred Reporting Items for Systematic Reviews and Meta-Analyses
PRISMA-ScR: Preferred Reporting Items for Systematic Reviews and Meta-Analyses Extension for Scoping Reviews
PRO: patient-reported outcome
QuADS: Quality Assessment for Diverse Studies
RPM: remote patient monitoring
TiDIER: Template for Intervention Description and Replication
