# Supplementary material for: Professional-Facing Digital Health Technology for the Care of Patients With Chronic Pain: Scoping Review
Source: J Med Internet Res. 2025 May 14;27:e66457. doi: 10.2196/66457 (PMC12120369; doi:10.2196/66457)
Supplement: Multimedia Appendix 3 [file jmir_v27i1e66457_app3.docx]

## Multimedia Appendix 3: Quality assessment results

Table 1: Table to show individual quality assessment scores using Qualitative Assessment of Diverse Studies (QuADS) for studies included in the scoping review, excluding descriptive reports of DHT (N=46)

| **Author** | **Criteria*** | | | | | | | | | | | | |  |
| --- | --- | --- | --- | --- | --- | --- | --- | --- | --- | --- | --- | --- | --- | --- |
|  | **1** | **2** | **3** | **4** | **5** | **6** | **7** | **8** | **9** | **10** | **11** | **12** | **13** | **Total** |
|  | ***Score***** | | | | | | | | | | | | |  |
| Allen et al (2022) [1] | 2 | 3 | 3 | 3 | 3 | 1 | 3 | 3 | 2 | 0 | 3 | 1 | 2 | 29 |
| Anderson et al (2016) [2] | 3 | 2 | 3 | 2 | 2 | 3 | 2 | 3 | 2 | 0 | 1 | 0 | 2 | 25 |
| Andrews et al (2022) [3] | 3 | 3 | 2 | 3 | 3 | 3 | 3 | 3 | 3 | 1 | 3 | 1 | 2 | 33 |
| Apathy et al (2022) [4] | 2 | 2 | 3 | 1 | 1 | 0 | 0 | 0 | 2 | 1 | 1 | 1 | 2 | 16 |
| Benavent et al (2022) [5] | 3 | 2 | 1 | 2 | 3 | 3 | 3 | 3 | 3 | 0 | 2 | 2 | 3 | 30 |
| Bernard et al (2022) [6] | 3 | 2 | 3 | 3 | 2 | 1 | 3 | 2 | 1 | 2 | 3 | 0 | 1 | 26 |
| Bhatia et al (2021) [7] | 1 | 3 | 2 | 3 | 2 | 3 | 3 | 2 | 2 | 0 | 3 | 0 | 2 | 26 |
| Butler et al (2016) [8] | 2 | 3 | 2 | 3 | 2 | 3 | 3 | 1 | 1 | 1 | 2 | 1 | 2 | 26 |
| Colomina et al (2021) [9] | 2 | 2 | 2 | 2 | 1 | 2 | 3 | 3 | 2 | 0 | 3 | 1 | 3 | 26 |
| Cordero-Tous et al (2022) [10] | 2 | 3 | 2 | 1 | 1 | 2 | 1 | 2 | 1 | 0 | 0 | 3 | 2 | 20 |
| Dhingra et al (2021) [11] | 2 | 1 | 2 | 2 | 2 | 0 | 3 | 2 | 3 | 0 | 3 | 1 | 2 | 23 |
| Dong et al (2014) [12] | 3 | 2 | 3 | 3 | 1 | 3 | 3 | 2 | 1 | 0 | 3 | 1 | 1 | 26 |
| Ekman et al (2020) [13] | 2 | 3 | 1 | 2 | 0 | 3 | 3 | 2 | 0 | 3 | 3 | 0 | 2 | 24 |
| Fanning et al (2020) [14] | 3 | 3 | 2 | 3 | 2 | 1 | 3 | 2 | 3 | 0 | 3 | 2 | 3 | 30 |
| Fedkov et al (2022) [15] | 3 | 3 | 1 | 2 | 3 | 0 | 2 | 1 | 3 | 1 | 2 | 1 | 3 | 25 |
| Goff et al (2023) [16] | 3 | 3 | 1 | 3 | 1 | 0 | 3 | 2 | 0 | 0 | 2 | 3 | 3 | 24 |
| Han et al (2022) [17] | 3 | 2 | 2 | 2 | 2 | 3 | 3 | 2 | 2 | 1 | 3 | 1 | 2 | 28 |
| Harle et al (2019) [18] | 2 | 3 | 2 | 3 | 1 | 1 | 0 | 1 | 1 | 0 | 1 | 2 | 3 | 20 |
| Ireland and Andrews (2019) [19] | 2 | 3 | 2 | 2 | 2 | 0 | 2 | 1 | 1 | 0 | 2 | 1 | 0 | 18 |
| Jansen-Kosterink et al (2021) [20] | 3 | 2 | 1 | 3 | 3 | 3 | 3 | 2 | 2 | 0 | 2 | 0 | 2 | 26 |
| Kampusch et al (2022) [21] | 1 | 2 | 1 | 3 | 0 | 0 | 0 | 1 | 1 | 0 | 0 | 0 | 0 | 9 |
| Kempin et al (2022) [22] | 2 | 2 | 1 | 2 | 1 | 0 | 1 | 2 | 3 | 0 | 2 | 0 | 2 | 18 |
| Kerckhove et al (2022) [23] | 2 | 3 | 3 | 2 | 2 | 0 | 2 | 2 | 2 | 0 | 3 | 0 | 3 | 24 |
| Klemm et al (2021) [24] | 2 | 1 | 1 | 2 | 1 | 3 | 2 | 0 | 2 | 0 | 2 | 1 | 2 | 19 |
| Knab et al (2001) [25] | 2 | 2 | 3 | 2 | 2 | 1 | 2 | 3 | 3 | 0 | 3 | 1 | 2 | 26 |
| Labinsky et al (2022) [26] | 2 | 2 | 2 | 3 | 1 | 1 | 2 | 1 | 0 | 0 | 1 | 1 | 2 | 18 |
| Lamper et al (2021) [27] | 3 | 2 | 3 | 3 | 2 | 1 | 3 | 1 | 0 | 0 | 3 | 1 | 2 | 24 |
| Li et al (2023) [28] | 2 | 2 | 2 | 3 | 3 | 0 | 3 | 2 | 3 | 0 | 3 | 0 | 2 | 25 |
| McCaffrey et al (2018) [29] | 1 | 3 | 2 | 2 | 2 | 2 | 2 | 1 | 1 | 3 | 3 | 0 | 0 | 22 |
| Muskens et al (2021) [30] | 2 | 3 | 3 | 3 | 2 | 0 | 3 | 1 | 1 | 0 | 3 | 0 | 1 | 22 |
| Neubert et al (2018) [31] | 1 | 2 | 3 | 2 | 2 | 0 | 2 | 2 | 2 | 0 | 2 | 1 | 2 | 21 |
| Papageorgiou et al (2021) [32] | 2 | 2 | 2 | 2 | 1 | 0 | 3 | 1 | 1 | 0 | 3 | 0 | 0 | 17 |
| Peiris et al (2014) [33] | 3 | 3 | 2 | 3 | 2 | 0 | 3 | 2 | 1 | 0 | 3 | 1 | 1 | 24 |
| Pers et al (2021) [34] | 3 | 2 | 1 | 3 | 3 | 0 | 3 | 3 | 3 | 1 | 3 | 0 | 2 | 27 |
| Price-Haywood et al (2018) [35] | 2 | 3 | 3 | 3 | 2 | 0 | 3 | 2 | 1 | 0 | 3 | 0 | 1 | 23 |
| Price-Haywood et al (2020) [36] | 2 | 3 | 2 | 3 | 1 | 0 | 3 | 2 | 1 | 1 | 3 | 0 | 1 | 22 |
| Selter et al (2018) [37] | 2 | 3 | 1 | 2 | 2 | 0 | 2 | 3 | 2 | 0 | 3 | 0 | 2 | 22 |
| Thomson et al (2020) [38] | 2 | 3 | 2 | 3 | 1 | 0 | 0 | 2 | 0 | 2 | 3 | 2 | 2 | 22 |
| Thomson et al (2021) [39] | 1 | 2 | 2 | 3 | 1 | 2 | 3 | 3 | 3 | 0 | 2 | 2 | 2 | 26 |
| Trafton et al (2010) [40] | 2 | 2 | 2 | 3 | 1 | 3 | 3 | 2 | 1 | 0 | 2 | 1 | 2 | 24 |
| van der Meer et al (2019) [41] | 2 | 2 | 3 | 3 | 2 | 2 | 2 | 3 | 1 | 0 | 2 | 0 | 3 | 25 |
| Verma et al (2014) [42] | 1 | 1 | 1 | 1 | 0 | 0 | 0 | 1 | 0 | 0 | 1 | 1 | 0 | 7 |
| Webers et al (2019) [43] | 3 | 3 | 2 | 3 | 3 | 0 | 0 | 2 | 1 | 0 | 2 | 1 | 0 | 20 |
| Yen et al (2016) [44] | 2 | 3 | 3 | 3 | 2 | 2 | 2 | 2 | 1 | 2 | 3 | 1 | 2 | 28 |
| Yin et al (2021) [45] | 1 | 2 | 1 | 2 | 0 | 0 | 2 | 2 | 0 | 0 | 0 | 0 | 1 | 11 |
| Zheng et al (2017) [46] | 2 | 2 | 2 | 2 | 1 | 0 | 0 | 2 | 1 | 0 | 1 | 1 | 1 | 15 |

**QuADS criteria: 1. theoretical or conceptual underpinning to the research; 2. statement of research aim/s; 3. clear description of research setting and target population; 4. the study design is appropriate to address the stated research aims; 5. appropriate sampling to address the research aim/s; 6. rationale for choice of data collection tool/s; 7. the format and content of data collection is appropriate to address the stated research aim/s; 8. description of data collection procedure; 9. recruitment data provided; 10. Justification for analytic method selected; 11. The method of analysis was appropriate to answer the research aim/s; 12. Evidence that the research stakeholders have been considered in research design or conduct; 13. Strengths and limitations critically discussed*

***QuADS score: ranges from 0-3, with 0 being low quality and 3 being high quality. QuADS does not have cut-off scores for quality assessment. Specific categorisation is presented in Table 2.*

Table 2: Table to show Quality Assessment for Diverse Studies (QuADS) criteria and specific categorisation of scores

| **QuADS criteria** | **0** | **1** | **2** | **3** | **Notes** |
| --- | --- | --- | --- | --- | --- |
| *1. Theoretical or conceptual underpinning to the research* | No mention at all. | General reference to broad theories or concepts that frame the study. e.g. key concepts were identified in the introduction section. | Identification of specific theories or concepts that frame the study and how these informed the work undertaken. e.g. key concepts were identified in the introduction section and applied to the study. | Explicit discussion of the theories or concepts that inform the study, with application of the theory or concept evident through the design, materials and outcomes explored. e.g. key concepts were identified in the introduction section and the application apparent in each element of the study design. |  |
| *2. Statement of research aim/s* | No mention at all. | Reference to what the sought to achieve embedded within the report but no explicit aims statement | Aims statement made but may only appear in the abstract or be lacking detail | Explicit and detailed statement of aim/s in the main body of report. |  |
| *3. Clear description of research setting and target population* | No mention at all. | General description of research area but not of the specific research environment e.g. ‘in primary care.’ | Description of research setting is made but is lacking detail e.g. ‘in primary care practices in region [x]’. | Specific description of the research setting and target population of study e.g. ‘nurses and doctors from GP practices in [x] part of [x] city in [x] country.’ |  |
| *4. The study design is appropriate to address the stated research aim/s* | No research aim/s stated or the design is entirely unsuitable e.g. a Y/N item survey for a study seeking to undertake exploratory work of lived experiences. | The study design can only address some aspects of the stated research aim/s e.g. use of focus groups to capture data regarding the frequency and experience of a disease. | The study design can address the stated research aim/s but there is a more suitable alternative that could have been used or used in addition e.g. addition of a qualitative or quantitative component could strengthen the design. | The study design selected appears to be the most suitable approach to attempt to answer the stated research aim/s. |  |
| *5. Appropriate sampling to address the research aim/s* | No mention of the sampling approach. | Evidence of consideration of the sample required e.g. the sample characteristics are described and appear appropriate to address the research aim/s. | Evidence of consideration of sample required to address the aim. e.g. the sample characteristics are described with reference to the aim/s | Detailed evidence of consideration of the sample required to address the research aim/s. e.g. sample size calculation or discussion of an iterative sampling process with reference to the research aims or the case selected for study. |  |
| *6. Rationale for choice of data collection tool/s* | No mention of rationale for data collection tool used. | Very limited explanation for choice of data collection tool/s. e.g. based on availability of tool. | Basic explanation of rationale for choice of data collection tool/s. e.g. based on use in a prior similar study. | Detailed explanation of rationale for choice of data collection tool/s. e.g. relevance to the study aim/s, codesigned with the target population or assessments of tool quality. |  |
| *7. The format and content of data collection tool is appropriate to address the stated research aim/s* | No research aim/s stated and/or data collection tool not detailed. | Structure and/or content of tool/s suitable to address some aspects of the research aim/s or to address the aim/s superficially e.g. single item response that is very general or an open-response item to capture content which requires probing. | Structure and/or content of tool/s allow for data to be gathered broadly addressing the stated aim/s but could benefit from refinement. e.g. the framing of survey or interview questions are too broad or focused to one element of the research aim/s. | Structure and content of tool/s allow for detailed data to be gathered around all relevant issues required to address the stated research aim/s |  |
| *8. Description of data collection procedure* | No mention of the data collection procedure. | Basic and brief outline of data collection procedure e.g. ‘using a questionnaire distributed to staff’. | States each stage of data collection procedure but with limited detail or states some stages in detail but omits others e.g. the recruitment process is mentioned but lacks important details. | Detailed description of each stage of the data collection procedure, including when, where and how data was gathered such that the procedure could be replicated. |  |
| *9. Recruitment data provided* | No mention of recruitment data. | Minimal and basic recruitment data e.g. number of people invited who agreed to take part. | Some recruitment data but not a complete account e.g. number of people who were invited and agreed. | Complete data allowing for full picture of recruitment outcomes e.g. number of people approached, recruited, and who completed with attrition data explained where relevant |  |
| *10. Justification for analytic method selected* | No mention of the rationale for the analytic method chosen | Very limited justification for choice of analytic method selected. e.g. previous use by the research team. | Basic justification for choice of analytic method selected e.g. method used in prior similar research. | Detailed justification for choice of analytic method selected e.g. relevance to the study aim/s or comment around of the strengths of the method selected. |  |
| *11. The method of analysis was appropriate to answer the research aim/s* | No mention at all. | Method of analysis can only address the research aim/s basically or broadly | Method of analysis can address the research aim/s but there is a more suitable alternative that could have been used or used in addition to offer a stronger analysis. | Method of analysis selected is the most suitable approach to attempt answer the research aim/s in detail e.g. for qualitative interpretative phenomenological analysis might be considered preferable for experiences vs. content analysis to elicit frequency of occurrence of events |  |
| *12. Evidence that the research stakeholders have been considered in research design or conduct.* | No mention at all. | Consideration of some the research stakeholders e.g. use of pilot study with target sample but no stakeholder involvement in planning stages of study design | Evidence of stakeholder input informing the research. e.g. use of pilot study with feedback influencing the study design/conduct or reference to a project reference group established to guide the research. | Substantial consultation with stakeholders identifiable in planning of study design and in preliminary work e.g. consultation in the conceptualisation of the research, a project advisory group or evidence of stakeholder input informing the work. |  |
| *13. Strengths and limitations critically discussed* | No mention at all. | Very limited mention of strengths and limitations with omissions of many key issues. e.g. one or two strengths/limitations mentioned with limited detail. | Discussion of some of the key strengths and weaknesses of the study but not complete. e.g. several strengths/limitations explored but with notable omissions or lack of depth of explanation. | Thorough discussion of strengths and limitations of all aspects of study including design, methods, data collection tools, sample & analytic approach |  |

QuADS not applied to following studies: Johnson et al (2021) [47]; Katzman et al (2013) [48]; Lin et al (2006) [49]; Pombo et al (2012) [50]; Trafton et al (2010) [51]; Shelley et al (2017) [52]

### References

1. Allen KS, Danielson, E. C., Downs, S. M., Mazurenko, O., Diiulio, J., Salloum, R. G., ... & Harle, C. A. . Evaluating a Prototype Clinical Decision Support Tool for Chronic Pain Treatment in Primary Care. *Applied Clinical Informatics* 2022;13(3):602-611. doi:10.1055/s-0042-1749332

2. Anderson DR, Zlateva I, Coman EN, Khatri K, Tian T, Kerns RD. Improving pain care through implementation of the Stepped Care Model at a multisite community health center. Report. *Journal of Pain Research*. 2016;9:1021. doi:10.2147/JPR.S117885

3. Andrews NE, Ireland D, Deen M, Varnfield M. Clinical utility of a mHealth assisted intervention for activity modulation in chronic pain: The pilot implementation of pain ROADMAP. *European Journal of Pain*. 2023;27(6):749-765. doi:10.1002/ejp.2104

4. Apathy NC, Sanner L, Adams MCB, et al. Assessing the use of a clinical decision support tool for pain management in primary care. *JAMIA Open*. 2022;5(3):ooac074. doi:10.1093/jamiaopen/ooac074

5. Benavent D, Fernández-Luque L, Núñez-Benjumea FJ, et al. Monitoring chronic inflammatory musculoskeletal diseases mixing virtual and face-to-face assessments—Results of the digireuma study. *PLOS Digital Health*. 2022;1(12):e0000157. doi:10.1371/journal.pdig.0000157

6. Bernard L, Valsecchi V, Mura T, et al. Management of patients with rheumatoid arthritis by telemedicine: connected monitoring. A randomized controlled trial. *Joint Bone Spine*. 2022;89(5):105368. doi:10.1016/j.jbspin.2022.105368

7. Bhatia A, Jamal K, Janmohamed T, et al. User Engagement and Clinical Impact of the Manage My Pain App in Patients With Chronic Pain: A Real-World, Multi-site Trial. *JMIR mHealth and uHealth*. 2021;9(3)doi:10.2196/26528

8. Butler SF, Zacharoff KL, Charity S, et al. Impact of an Electronic Pain and Opioid Risk Assessment Program: Are There Improvements in Patient Encounters and Clinic Notes? Article. *Pain Medicine*. 2016;17:2047+. doi:10.1093/pm/pnw033

9. Colomina J, Reis D, Torra M, et al. Implementing mHealth-Enabled Integrated Care for Complex Chronic Patients With Osteoarthritis Undergoing Primary Hip or Knee Arthroplasty: Prospective, Two-Arm, Parallel Trial. *Journal of Medical Internet Research*. 2021;doi:10.2196/28320

10. Cordero Tous N, Santos Martín L, Sánchez Corral C, et al. Development of an integrated solution for patients with neurostimulator for chronic pain in times of COVID-19: A mobile application with a support center. *Neurocirugía (English Edition)*. 2022;33(6):318-327. doi:10.1016/j.neucie.2021.12.001

11. Dhingra L, Schiller R, Teets R, et al. Pain Management in Primary Care: A Randomized Controlled Trial of a Computerized Decision Support Tool. *The American Journal of Medicine*. 2021;134(12):1546-1554. doi:10.1016/j.amjmed.2021.07.014

12. Dong Z, Yin Z, He M, Chen X, Lv X, Yu S. Validation of a guideline-based decision support system for the diagnosis of primary headache disorders based on ICHD-3 beta. *The journal of headache and pain*. 2014;15:40. doi:10.1186/1129-2377-15-40

13. Ekman B, Nero H, Lohmander LS, Dahlberg LE. Costing analysis of a digital first-line treatment platform for patients with knee and hip osteoarthritis in Sweden. Report. *PLoS ONE*. 2020;15:e0236342. doi:10.1371/journal.pone.0236342

14. Fanning J, Brooks AK, Ip E, et al. A Mobile Health Behavior Intervention to Reduce Pain and Improve Health in Older Adults With Obesity and Chronic Pain: The MORPH Pilot Trial. Clinical Trial. *Frontiers in Digital Health*. 2020;2doi:10.3389/fdgth.2020.598456

15. Fedkov D, Berghofen A, Weiss C, et al. Efficacy and safety of a mobile app intervention in patients with inflammatory arthritis: a prospective pilot study. *Rheumatology International*. 2022;42(12):2177-2190. doi:10.1007/s00296-022-05175-4

16. Goff AJ, De Oliveira Silva D, Ezzat AM, Crossley KM, Pazzinatto MF, Barton CJ. Co-design of the web-based ‘My Knee’ education and self-management toolkit for people with knee osteoarthritis. *Digital Health*. 2023;9doi:10.1177/20552076231163810

17. Han JJ, Graham JH, Snyder DI, Alfieri T. Long-term Use of Wearable Health Technology by Chronic Pain Patients. *The Clinical journal of pain*. 2022;38(12):701-710. doi:10.1097/AJP.0000000000001076

18. Harle CA, DiIulio J, Downs SM, et al. Decision-Centered Design of Patient Information Visualizations to Support Chronic Pain Care. *Applied clinical informatics*. 2019;10(4):719-728. doi:10.1055/s-0039-1696668

19. Ireland D, Andrews N. Pain ROADMAP: A mobile platform to support activity pacing for chronic pain. In: Ebooks IP, ed. *Digital health: changing the way healthcare is conceptualised and delivered*. 2019:89-94.

20. Jansen-Kosterink S, van Velsen L, Cabrita M. Clinician acceptance of complex clinical decision support systems for treatment allocation of patients with chronic low back pain. *BMC medical informatics and decision making*. 2021;21(1):137-137. doi:10.1186/s12911-021-01502-0

21. Kampusch S, Edegger K, Mayr P, et al. Integrated Platform for the Management of Chronic Low Back Pain. In: Press I, ed. *dHealth*. 2022:260-261.

22. Kempin R, Richter JG, Schlegel A, et al. Monitoring of Disease Activity With a Smartphone App in Routine Clinical Care in Patients With Axial Spondyloarthritis. *Journal of rheumatology*. 2022;49(8):878-884. doi:10.3899/jrheum.211116

23. Kerckhove N, Delage N, Cambier S, et al. eDOL mHealth App and Web Platform for Self-monitoring and Medical Follow-up of Patients With Chronic Pain: Observational Feasibility Study. *JMIR formative research*. 2022;6(3):e30052-e30052. doi:10.2196/30052

24. Klemm P, Kleyer A, Tascilar K, et al. A virtual reality based app to educate health care professionals and medical students about inflammatory arthritis: Feasibility study. *JMIR serious games*. 2021;9(2):e23835-e23835. doi:10.2196/23835

25. Knab JH, Wallace MS, Wagner RL, Tsoukatos J, Weinger MB. The Use of a Computer-Based Decision Support System Facilitates Primary Care Physicians’ Management of Chronic Pain. *Anesthesia & Analgesia*. 2001;93(3):712-720. doi:10.1097/00000539-200109000-00035

26. Labinsky H, Ukalovic D, Hartmann F, et al. An AI-Powered Clinical Decision Support System to Predict Flares in Rheumatoid Arthritis: A Pilot Study. *Diagnostics (Basel)*. 2023;13(1):148. doi:10.3390/diagnostics13010148

27. Lamper C, Huijnen I, Mooij Md, Köke A, Verbunt J, Kroese M. An ecoach-pain for patients with chronic musculoskeletal pain in interdisciplinary primary care: A feasibility study. *International journal of environmental research and public health*. 2021;18(21):11661. doi:10.3390/ijerph182111661

28. Li C, Huang J, Wu H, et al. Management of Rheumatoid Arthritis with a Digital Health Application: A Multicenter, Pragmatic Randomized Clinical Trial. *JAMA network open*. 2023;6(4):E238343-e238343. doi:10.1001/jamanetworkopen.2023.8343

29. McCaffrey SA, Black RA, Butler SF. Psychometric evaluation of the PainCAS Interference with Daily Activities, Psychological/Emotional Distress, and Pain scales. *Quality of life research*. 2018;27(3):835-843. doi:10.1007/s11136-017-1766-3

30. Müskens WD, Rongen-van Dartel SAA, Vogel C, Huis A, Adang EMM, van Riel PLCM. Telemedicine in the management of rheumatoid arthritis: maintaining disease control with less health-care utilization. *Rheumatology advances in practice*. 2021;5(1):rkaa079-rkaa079. doi:10.1093/rap/rkaa079

31. Neubert TA, Dusch M, Karst M, Beissner F. Designing a tablet-based software app for mapping bodily symptoms: Usability evaluation and reproducibility analysis. *JMIR mHealth and uHealth*. 2018;6(5):e127-e127. doi:10.2196/mhealth.8409

32. Papageorgiou L, Zervou MI, Vlachakis D, et al. Demetra Application: An integrated genotype analysis web server for clinical genomics in endometriosis. *International journal of molecular medicine*. 2021;47(6):1. doi:10.3892/ijmm.2021.4948

33. Peiris D, Williams C, Holbrook R, et al. A web-based clinical decision support tool for primary health care management of back pain: Development and mixed methods evaluation. *JMIR research protocols*. 2014;3(2):e17-e17. doi:10.2196/resprot.3071

34. Pers Y-M, Valsecchi V, Mura T, et al. A randomized prospective open-label controlled trial comparing the performance of a connected monitoring interface versus physical routine monitoring in patients with rheumatoid arthritis. *Rheumatology*. 2021;60(4):1659-1668. doi:10.1093/rheumatology/keaa462

35. Price-Haywood EG, Robinson W, Harden-Barrios J, Burton J, Burstain T. Intelligent clinical decision support to improve safe opioid management of chronic noncancer pain in primary care. *The Ochsner journal*. 2018;18(1):30-35. doi:10.1043/TOJ-17-0093

36. Price-Haywood EG, Burton J, Burstain T, et al. Clinical Effectiveness of Decision Support for Prescribing Opioids for Chronic Noncancer Pain: A Prospective Cohort Study. *Value in health*. 2020;23(2):157-163. doi:10.1016/j.jval.2019.09.2748

37. Selter A, Tsangouri C, Ali SB, et al. An mHealth app for self-management of chronic lower back pain (Limbr): Pilot study. *JMIR mHealth and uHealth*. 2018;20(9):e179-e179. doi:10.2196/mhealth.8256

38. Thomson S, Huygen F, Prangnell S, et al. Appropriate referral and selection of patients with chronic pain for spinal cord stimulation: European consensus recommendations and e‐health tool. *European journal of pain*. 2020;24(6):1169-1181. doi:10.1002/ejp.1562

39. Thomson S, Huygen F, Prangnell S, et al. Applicability and Validity of an e-Health Tool for the Appropriate Referral and Selection of Patients With Chronic Pain for Spinal Cord Stimulation: Results From a European Retrospective Study. *Neuromodulation: Technology at the Neural Interface*. 2023;26(1):164-171. doi:10.1016/j.neurom.2021.12.006

40. Trafton J, Martins S, Michel M, et al. Evaluation of the Acceptability and Usability of a Decision Support System to Encourage Safe and Effective Use of Opioid Therapy for Chronic, Noncancer Pain by Primary Care Providers. *Pain medicine (Malden, Mass)*. 2010;11(4):575-585. doi:10.1111/j.1526-4637.2010.00818.x

41. van der Meer HA, Doomen A, Visscher CM, Engelbert RHH, Nijhuis-van der Sanden MWG, Speksnijder CM. The additional value of e-Health for patients with a temporomandibular disorder: a mixed methods study on the perspectives of orofacial physical therapists and patients. *Disability and rehabilitation: Assistive technology*. 2022;1-13. doi:10.1080/17483107.2022.2094000

42. Verma SK, Chun S, Liu BJ. A web-based neurological pain classifier tool utilizing Bayesian decision theory for pain classification in spinal cord injury patients. SPIE; 2014:90390E-90390E-8.

43. Webers C, Beckers E, Boonen A, et al. Development, usability and acceptability of an integrated eHealth system for spondyloarthritis in the Netherlands (SpA-Net). *Rheumatic & musculoskeletal diseases open*. 2019;5(1):e000860-e000860. doi:10.1136/rmdopen-2018-000860

44. Yen PY, Lara B, Lopetegui M, et al. Usability and workflow evaluation of “RhEumAtic disease activity” (READY): A mobile application for rheumatology patients and providers. *Applied clinical informatics*. 2016;7(4):1007-1024. doi:10.4338/ACI-2016-03-RA-0036

45. Yin Z, Zhou L, He M, Chen X. MyHeadache: an intelligent headache diary mobile application to enhance patient compliance. IEEE; 2021:1-8.

46. Zheng H, Tulu B, Choi W, Franklin P. Using mHealth App to Support Treatment Decision-Making for Knee Arthritis: Patient Perspective. *EGEMS (Washington, DC)*. 2017;5(2):7-7. doi:10.13063/2327-9214.1284

47. Johnson CB. A Personalized Shared Decision-Making Tool for Osteoarthritis Management of the Knee. *Orthopaedic Nursing*. 2021;40(2)doi:10.1097/NOR.0000000000000739

48. Katzman JG. Making Connections: Using TeleHealth to Improve the Diagnosis and Treatment of Complex Regional Pain Syndrome, an Underrecognized Neuroinflammatory Disorder. *Journal of Neuroimmune Pharmacology*. 2013;8(3):489-493. doi:10.1007/s11481-012-9408-6

49. Lin L, Hu PJ-H, Liu Sheng OR. A decision support system for lower back pain diagnosis: Uncertainty management and clinical evaluations. *DECISION SUPPORT SYSTEMS*. 2006;42(2):1152-1169. doi:10.1016/j.dss.2005.10.007

50. Pombo N, Araújo P, Viana J, Junior B, Serrano R. Contribution of web services to improve pain diaries experience. Int Assoc Engineers-Iaeng; 2012; 589-592.

51. Trafton JA, Martins SB, Michel MC, et al. Designing an automated clinical decision support system to match clinical practice guidelines for opioid therapy for chronic pain. Report. *Implementation Science*. 2010;5:26. doi:10.1186/1748-5908-5-26

52. Shelley BM, Katzman JG, Comerci GD, et al. ECHO pain curriculum: Balancing mandated continuing education with the needs of rural health care practitioners. *The Journal of continuing education in the health professions*. 2017;37(3):190-194. doi:10.1097/CEH.0000000000000165
