# Supplementary material for: Professional-Facing Digital Health Technology for the Care of Patients With Chronic Pain: Scoping Review
Source: J Med Internet Res. 2025 May 14;27:e66457. doi: 10.2196/66457 (PMC12120369; doi:10.2196/66457)
Supplement: Multimedia Appendix 4 [file jmir_v27i1e66457_app4.docx]

## Multimedia Appendix 4: Table of included study characteristics (N=52)

Table 1: Table describing the characteristics of studies included in the scoping review (N=52)

| **Author**  **(year)** | **Country** | **Study design** | **Target CP**  **condition** | **Target population (HCP)** |  |  |  | **Setting** |
| --- | --- | --- | --- | --- | --- | --- | --- | --- |
|  |  |  |  | *Profession* | *N* | *Sex* | *Experience (mean years)* |  |
| Allen et al (2022) [1] | USA | Interview study | Chronic pain (non-cancer) | PCP (family medicine or internal medicine doctors) | 12 | 6 m, 6 f | N/A | 10 primary care clinics |
| Anderson et al (2016) [2] | USA | Retrospective data, chart review and survey study | Chronic pain | PCP (family medicine or internal medicine doctors, family nurse practitioners, physician assistants) | 25 | 11 m, 14 f | N/A | 12 primary care clinics |
| Andrews et al (2023) [3] | Australia | Pilot study | Chronic pain (non-cancer) | Therapist | 1 | 1 f | N/A | 1 outpatient clinic at multidisciplinary pain centre |
| Apathy et al (2022) [4] | USA | Retrospective analysis | Chronic pain (non-cancer) | PCP (medical or osteopathic physicians) | 69 | 48 f,  21 m | 10 years (SD= 9.9) | 2 health centres |
| Benavent et al (2022) [5] | Australia | Prospective usability and feasibility study | RA and SpA | Clinician/ rheumatologist | 2 in focus group; 2 in pilot study | N/A | N/A | Complex therapy unit |
| Bernard et al (2022) [6] | France | Randomised controlled trial | RA | Rheumatologist | N/A | N/A | N/A | Rheumatology department of hospital |
| Bhatia et al (2021) [7] | Canada | Prospective exploratory study | Chronic pain | Clinician | N/A | N/A | N/A | 3 pain clinics |
| Butler et al (2016) [8] | USA | Multi-phase and mixed-method (chart review, interview and questionnaire) study | Chronic pain | Physician; nurse practitioner; psychologist | 23 in study 1; 18 in study 2 | N/A | 20 years of clinical experience, 12 years in chronic pain care | 3 pain clinics |
| Colomina et al (2021) [9] | Spain | Prospective, two-arm, parallel trial | Osteoarthritis (with hip or knee arthroplasty) | Family physicians, hospital surgical team and social workers | N/A | N/A | N/A | University hospital and primary care centre |
| Cordero-Tous et al (2022) [10] | Spain | Multi-phase, mixed-method study | Chronic pain | Anaesthetist; physicians; neurophysiologists; neurologists; neurosurgeons; clinical professionals | 5 in phase 1 | N/A | N/A | Remote |
| Dhingra et al (2021) [11] | USA | Randomised controlled trial | Chronic pain | PCPs | N/A | N/A | N/A | 6 primary care practices |
|  |  |  |  |  |  |  |  |  |
| Dong et al (2014) [12] | China | Prospective validation study | Primary headache (inc. chronic headache) | General physicians | N/A | N/A | N/A | Headache centre, General hospital |
| Ekman et al (2020) [13] | Sweden | Costing analysis | Knee and hip osteoarthritis | Physiotherapists or other HCP | N/A | N/A | N/A | N/A |
| Fanning et al (2020) [14] | USA | Pilot study | Chronic pain | Behavioural interventionists and nutritionists | N/A | N/A | N/A | Pain clinics |
| Fedkov et al (2022) [15] | Finland | Pilot study | Rheumatoid arthritis, spondylarthritis and psoriatic arthritis | Physicians | N/A | N/A | N/A | Remote |
| Goff et al (2023) [16] | Singapore | Multi-phase co-design study | Knee osteoarthritis | Physiotherapists, exercise physiologists, nurse practitioners | 9 | 8 f, 1 m | 17 years (5-24) | N/A |
| Han et al (2022) [17] | USA | Prospective observational study | Chronic pain | Physicians, physician assistants and nurse practitioners | N/A | N/A | N/A | 1 pain department of hospital |
| Harle et al (2019) [18] | USA | Interview study | Chronic pain (non-cancer) | PCPs (physicians and nurse practitioners) | 10 in Phase 1 and 14 in Phase 2 | 5 f, 5 m | 15 years | 4 primary care clinics |
| Ireland and Andrews (2019) [19] | Australia | Pilot study | Chronic pain | Clinicians | N/A | N/A | N/A | Hospital |
| Jansen-Kosterink et al (2021) [20] | The Netherlands | Questionnaire study | Chronic low back pain | GPs, primary care physiotherapists and other HCPs | 98 | 47 f, 51 m | Less than a year (1%); 1-5 years (10%); 6-10 years (17%); 11-20 years (24%); >21 years (48%) | Primary care centre |
| Johnson (2021) [21] | USA | Description of intervention | Osteoarthritis | HCPs | N/A | N/A | N/A | Primary care centre |
|  |  |  |  |  |  |  |  |  |
| Katzman (2013) [22] | USA | Description of intervention | Complex regional pain syndrome | Interdisciplinary clinicians | N/A | N/A | N/A | N/A |
| Kampusch et al (2022) [23] | Austria | Prospective exploratory study | Chronic low back pain | Clinicians | N/A | N/A | N/A | N/A |
| Kempin et al (2022) [24] | Germany | Single-centre longitudinal study | Axial spondylarthritis | Rheumatologists and physicians | N/A | N/A | N/A | Rheumatology centre |
| Kerkhove et al (2022) [25] | France | Observational feasibility study | Chronic pain | Physicians | N/A | N/A | N/A | 12 pain clinics |
| Klemm et al (2021) [26] | Germany | Feasibility study | Inflammatory arthritis | Physicians and other HCPs | 125 | 70 f, 54 m, 1 non-binary | N/A | N/A |
| Knab et al (2001) [27] | USA | Prospective exploratory study | Chronic pain | PCPs (internists and gynaecologist) | 5 | N/A | N/A | Pain clinic |
| Labinsky et al (2023) [28] | Germany | Pilot study | Rheumatoid arthritis | Physicians | 5 | 3 f, 2 m | 1-5 years | Rheumatology outpatient clinic |
| Lamper et al (2021) [29] | The Netherlands | Feasibility study | Musculoskeletal pain | PCPs (GPs, practice nurses, physiotherapists) | 6 | N/A | N/A | 2 primary care practices |
| Li et al (2023) [30] | China | Randomised controlled trial | Rheumatoid arthritis | Rheumatologists | N/A | N/A | N/A | 22 hospitals |
| Lin et al (2006) [31] | USA | Prospective exploratory study | Chronic low back pain | Clinician | N/A | N/A | N/A | N/A |
| McCaffrey et al (2018) [32] | USA | Cross-sectional exploratory study | Chronic pain | Clinician | 5 | N/A | N/A | 16 pain treatment centres |
| Muskens et al (2021) [33] | The Netherlands | Observational study | Rheumatoid arthritis | Rheumatologists and other HCPs (nurses, GPs, physiotherapists) | N/A | N/A | N/A | Rheumatology clinic |
| Neubert et al (2018) [34] | Germany | Multi-phase usability and evaluation study | Chronic pain | Anaesthiologists | 6 (study 1); 4 (study 2) | N/A | 5 years or more | Pain outpatient department |
| Papageorgiou et al (2021) [35] | Greece | Developmental study | Endometriosis | Medical experts and health experts | N/A | N/A | N/A | General hospital |
| Peiris et al (2014) [36] | Australia | Mixed-methods study | Chronic low back pain | GPs | 20 | 7 f, 13 m | N/A | 20 GP practices |
| Pers et al (2021) [37] | France | Randomised controlled trial | Rheumatoid arthritis | Physicians, rheumatologists and clinical nurse manager | N/A | N/A | N/A | Hospital |
| Pombo et al (2012) [38] | Portugal | Description of intervention | Chronic pain | Physician/HCP | N/A | N/A | N/A | N/A |
| Price-Haywood et al (2018) [39] | USA | Prospective cohort study | Chronic pain (non-cancer) | PCPs (internal medicine and family medicine) | N/A | N/A | N/A | 36 primary care clinics |
| Price-Haywood et al (2020) [40] | USA | Prospective cohort study | Chronic pain (non-cancer) | PCPs (internal medicine and family medicine) | N/A | N/A | N/A | 36 primary care clinics |
| Selter et al (2018) [41] | USA | Pilot study | Chronic low back pain | Certified health coach | N/A | N/A | N/A | N/A |
| Shelley et al (2017) [42] | USA | Description of intervention | Chronic pain | Chronic pain practitioners | N/A | N/A | N/A | N/A |
| Thomson et al (2020) [43] | Belgium | Modified Delphi study | Chronic pain | HCPs (referrers and implanters) | 18 | N/A | N/A | 12 implant centres |
| Thomson et al (2023) [44] | Multiple | Retrospective study | Chronic pain | HCPs | 18 | N/A | N/A | 12 implant centres |
| Trafton et al (2010) [45] | USA | Multi-phase usability and acceptability study | Chronic pain (non-cancer) | PCPs (psychiatrists, physicians, nurse practitioners) | Simulation testing - 4 (phase 1); 5 (phase 2); clinic testing - 10 (phase 1); 2 (phase 2) | N/A | N/A | 12 primary care clinics |
| Trafton et al (2010) [46] | USA | Developmental study | Chronic pain (non-cancer) | PCPs (psychiatrists. Physicians, nurse practitioners) | N/A | N/A | N/A | Pain management clinic |
| van der Meer et al (2022) [47] | The Netherlands | Mixed-methods study | Temporomandibular disorder | Specialised orofacial physical therapists | 10 | 2 f, 8 m | 11.5 years | Physical therapy centre |
| Verma et al (2014) [48] | USA | Mixed-methods study | Chronic pain | Clinician/physicians | N/A | N/A | N/A | N/A |
| Webers et al (2019) [49] | The Netherlands | Multi-phase usability and acceptability study | Spondylarthritis | Rheumatologists and nurses | 16 | N/A | N/A | 5 rheumatology centres |
| Yen et al (2016) [50] | USA | Cross-sectional usability and time motion study | Rheumatoid arthritis | Physicians | 15 | N/A | N/A | 2 outpatient clinics |
| Yin et al (2021) [51] | China | Mixed-method study | Chronic headache | Physicians | N/A | N/A | N/A | General hospital |
| Zheng et al (2017) [52] | USA | Interview and focus group study | Knee arthritis | Physiotherapists and knee replacement surgeons | 3 | N/A | 1 junior; 2 with 20 or more years | Arthritis and joint centre |

^a^ PCP = primary care professional; HCP = healthcare professional; N/A = not applicable, as study did not report or was not relevant

### References

1. Allen KS, Danielson, E. C., Downs, S. M., Mazurenko, O., Diiulio, J., Salloum, R. G., ... & Harle, C. A. . Evaluating a Prototype Clinical Decision Support Tool for Chronic Pain Treatment in Primary Care. *Applied Clinical Informatics* 2022;13(3):602-611. doi:10.1055/s-0042-1749332

2. Anderson DR, Zlateva I, Coman EN, Khatri K, Tian T, Kerns RD. Improving pain care through implementation of the Stepped Care Model at a multisite community health center. Report. *Journal of Pain Research*. 2016;9:1021. doi:10.2147/JPR.S117885

3. Andrews NE, Ireland D, Deen M, Varnfield M. Clinical utility of a mHealth assisted intervention for activity modulation in chronic pain: The pilot implementation of pain ROADMAP. *European Journal of Pain*. 2023;27(6):749-765. doi:10.1002/ejp.2104

4. Apathy NC, Sanner L, Adams MCB, et al. Assessing the use of a clinical decision support tool for pain management in primary care. *JAMIA Open*. 2022;5(3):ooac074. doi:10.1093/jamiaopen/ooac074

5. Benavent D, Fernández-Luque L, Núñez-Benjumea FJ, et al. Monitoring chronic inflammatory musculoskeletal diseases mixing virtual and face-to-face assessments—Results of the digireuma study. *PLOS Digital Health*. 2022;1(12):e0000157. doi:10.1371/journal.pdig.0000157

6. Bernard L, Valsecchi V, Mura T, et al. Management of patients with rheumatoid arthritis by telemedicine: connected monitoring. A randomized controlled trial. *Joint Bone Spine*. 2022;89(5):105368. doi:10.1016/j.jbspin.2022.105368

7. Bhatia A, Jamal K, Janmohamed T, et al. User Engagement and Clinical Impact of the Manage My Pain App in Patients With Chronic Pain: A Real-World, Multi-site Trial. *JMIR mHealth and uHealth*. 2021;9(3)doi:10.2196/26528

8. Butler SF, Zacharoff KL, Charity S, et al. Impact of an Electronic Pain and Opioid Risk Assessment Program: Are There Improvements in Patient Encounters and Clinic Notes? Article. *Pain Medicine*. 2016;17:2047+. doi:10.1093/pm/pnw033

9. Colomina J, Reis D, Torra M, et al. Implementing mHealth-Enabled Integrated Care for Complex Chronic Patients With Osteoarthritis Undergoing Primary Hip or Knee Arthroplasty: Prospective, Two-Arm, Parallel Trial. *Journal of Medical Internet Research*. 2021;doi:10.2196/28320

10. Cordero Tous N, Santos Martín L, Sánchez Corral C, et al. Development of an integrated solution for patients with neurostimulator for chronic pain in times of COVID-19: A mobile application with a support center. *Neurocirugía (English Edition)*. 2022;33(6):318-327. doi:10.1016/j.neucie.2021.12.001

11. Dhingra L, Schiller R, Teets R, et al. Pain Management in Primary Care: A Randomized Controlled Trial of a Computerized Decision Support Tool. *The American Journal of Medicine*. 2021;134(12):1546-1554. doi:10.1016/j.amjmed.2021.07.014

12. Dong Z, Yin Z, He M, Chen X, Lv X, Yu S. Validation of a guideline-based decision support system for the diagnosis of primary headache disorders based on ICHD-3 beta. *The journal of headache and pain*. 2014;15:40. doi:10.1186/1129-2377-15-40

13. Ekman B, Nero H, Lohmander LS, Dahlberg LE. Costing analysis of a digital first-line treatment platform for patients with knee and hip osteoarthritis in Sweden. Report. *PLoS ONE*. 2020;15:e0236342. doi:10.1371/journal.pone.0236342

14. Fanning J, Brooks AK, Ip E, et al. A Mobile Health Behavior Intervention to Reduce Pain and Improve Health in Older Adults With Obesity and Chronic Pain: The MORPH Pilot Trial. Clinical Trial. *Frontiers in Digital Health*. 2020;2doi:10.3389/fdgth.2020.598456

15. Fedkov D, Berghofen A, Weiss C, et al. Efficacy and safety of a mobile app intervention in patients with inflammatory arthritis: a prospective pilot study. *Rheumatology International*. 2022;42(12):2177-2190. doi:10.1007/s00296-022-05175-4

16. Goff AJ, De Oliveira Silva D, Ezzat AM, Crossley KM, Pazzinatto MF, Barton CJ. Co-design of the web-based ‘My Knee’ education and self-management toolkit for people with knee osteoarthritis. *Digital Health*. 2023;9doi:10.1177/20552076231163810

17. Han JJ, Graham JH, Snyder DI, Alfieri T. Long-term Use of Wearable Health Technology by Chronic Pain Patients. *The Clinical journal of pain*. 2022;38(12):701-710. doi:10.1097/AJP.0000000000001076

18. Harle CA, DiIulio J, Downs SM, et al. Decision-Centered Design of Patient Information Visualizations to Support Chronic Pain Care. *Applied clinical informatics*. 2019;10(4):719-728. doi:10.1055/s-0039-1696668

19. Ireland D, Andrews N. Pain ROADMAP: A mobile platform to support activity pacing for chronic pain. In: Ebooks IP, ed. *Digital health: changing the way healthcare is conceptualised and delivered*. 2019:89-94.

20. Jansen-Kosterink S, van Velsen L, Cabrita M. Clinician acceptance of complex clinical decision support systems for treatment allocation of patients with chronic low back pain. *BMC medical informatics and decision making*. 2021;21(1):137-137. doi:10.1186/s12911-021-01502-0

21. Johnson CB. A Personalized Shared Decision-Making Tool for Osteoarthritis Management of the Knee. *Orthopaedic Nursing*. 2021;40(2)doi:10.1097/NOR.0000000000000739

22. Katzman JG. Making Connections: Using TeleHealth to Improve the Diagnosis and Treatment of Complex Regional Pain Syndrome, an Underrecognized Neuroinflammatory Disorder. *Journal of Neuroimmune Pharmacology*. 2013;8(3):489-493. doi:10.1007/s11481-012-9408-6

23. Kampusch S, Edegger K, Mayr P, et al. Integrated Platform for the Management of Chronic Low Back Pain. In: Press I, ed. *dHealth*. 2022:260-261.

24. Kempin R, Richter JG, Schlegel A, et al. Monitoring of Disease Activity With a Smartphone App in Routine Clinical Care in Patients With Axial Spondyloarthritis. *Journal of rheumatology*. 2022;49(8):878-884. doi:10.3899/jrheum.211116

25. Kerckhove N, Delage N, Cambier S, et al. eDOL mHealth App and Web Platform for Self-monitoring and Medical Follow-up of Patients With Chronic Pain: Observational Feasibility Study. *JMIR formative research*. 2022;6(3):e30052-e30052. doi:10.2196/30052

26. Klemm P, Kleyer A, Tascilar K, et al. A virtual reality based app to educate health care professionals and medical students about inflammatory arthritis: Feasibility study. *JMIR serious games*. 2021;9(2):e23835-e23835. doi:10.2196/23835

27. Knab JH, Wallace MS, Wagner RL, Tsoukatos J, Weinger MB. The Use of a Computer-Based Decision Support System Facilitates Primary Care Physicians’ Management of Chronic Pain. *Anesthesia & Analgesia*. 2001;93(3):712-720. doi:10.1097/00000539-200109000-00035

28. Labinsky H, Ukalovic D, Hartmann F, et al. An AI-Powered Clinical Decision Support System to Predict Flares in Rheumatoid Arthritis: A Pilot Study. *Diagnostics (Basel)*. 2023;13(1):148. doi:10.3390/diagnostics13010148

29. Lamper C, Huijnen I, Mooij Md, Köke A, Verbunt J, Kroese M. An ecoach-pain for patients with chronic musculoskeletal pain in interdisciplinary primary care: A feasibility study. *International journal of environmental research and public health*. 2021;18(21):11661. doi:10.3390/ijerph182111661

30. Li C, Huang J, Wu H, et al. Management of Rheumatoid Arthritis with a Digital Health Application: A Multicenter, Pragmatic Randomized Clinical Trial. *JAMA network open*. 2023;6(4):E238343-e238343. doi:10.1001/jamanetworkopen.2023.8343

31. Lin L, Hu PJ-H, Liu Sheng OR. A decision support system for lower back pain diagnosis: Uncertainty management and clinical evaluations. *DECISION SUPPORT SYSTEMS*. 2006;42(2):1152-1169. doi:10.1016/j.dss.2005.10.007

32. McCaffrey SA, Black RA, Butler SF. Psychometric evaluation of the PainCAS Interference with Daily Activities, Psychological/Emotional Distress, and Pain scales. *Quality of life research*. 2018;27(3):835-843. doi:10.1007/s11136-017-1766-3

33. Müskens WD, Rongen-van Dartel SAA, Vogel C, Huis A, Adang EMM, van Riel PLCM. Telemedicine in the management of rheumatoid arthritis: maintaining disease control with less health-care utilization. *Rheumatology advances in practice*. 2021;5(1):rkaa079-rkaa079. doi:10.1093/rap/rkaa079

34. Neubert TA, Dusch M, Karst M, Beissner F. Designing a tablet-based software app for mapping bodily symptoms: Usability evaluation and reproducibility analysis. *JMIR mHealth and uHealth*. 2018;6(5):e127-e127. doi:10.2196/mhealth.8409

35. Papageorgiou L, Zervou MI, Vlachakis D, et al. Demetra Application: An integrated genotype analysis web server for clinical genomics in endometriosis. *International journal of molecular medicine*. 2021;47(6):1. doi:10.3892/ijmm.2021.4948

36. Peiris D, Williams C, Holbrook R, et al. A web-based clinical decision support tool for primary health care management of back pain: Development and mixed methods evaluation. *JMIR research protocols*. 2014;3(2):e17-e17. doi:10.2196/resprot.3071

37. Pers Y-M, Valsecchi V, Mura T, et al. A randomized prospective open-label controlled trial comparing the performance of a connected monitoring interface versus physical routine monitoring in patients with rheumatoid arthritis. *Rheumatology*. 2021;60(4):1659-1668. doi:10.1093/rheumatology/keaa462

38. Pombo N, Araújo P, Viana J, Junior B, Serrano R. Contribution of web services to improve pain diaries experience. Int Assoc Engineers-Iaeng; 2012; 589-592.

39. Price-Haywood EG, Robinson W, Harden-Barrios J, Burton J, Burstain T. Intelligent clinical decision support to improve safe opioid management of chronic noncancer pain in primary care. *The Ochsner journal*. 2018;18(1):30-35. doi:10.1043/TOJ-17-0093

40. Price-Haywood EG, Burton J, Burstain T, et al. Clinical Effectiveness of Decision Support for Prescribing Opioids for Chronic Noncancer Pain: A Prospective Cohort Study. *Value in health*. 2020;23(2):157-163. doi:10.1016/j.jval.2019.09.2748

41. Selter A, Tsangouri C, Ali SB, et al. An mHealth app for self-management of chronic lower back pain (Limbr): Pilot study. *JMIR mHealth and uHealth*. 2018;20(9):e179-e179. doi:10.2196/mhealth.8256

42. Shelley BM, Katzman JG, Comerci GD, et al. ECHO pain curriculum: Balancing mandated continuing education with the needs of rural health care practitioners. *The Journal of continuing education in the health professions*. 2017;37(3):190-194. doi:10.1097/CEH.0000000000000165

43. Thomson S, Huygen F, Prangnell S, et al. Appropriate referral and selection of patients with chronic pain for spinal cord stimulation: European consensus recommendations and e‐health tool. *European journal of pain*. 2020;24(6):1169-1181. doi:10.1002/ejp.1562

44. Thomson S, Huygen F, Prangnell S, et al. Applicability and Validity of an e-Health Tool for the Appropriate Referral and Selection of Patients With Chronic Pain for Spinal Cord Stimulation: Results From a European Retrospective Study. *Neuromodulation: Technology at the Neural Interface*. 2023;26(1):164-171. doi:10.1016/j.neurom.2021.12.006

45. Trafton J, Martins S, Michel M, et al. Evaluation of the Acceptability and Usability of a Decision Support System to Encourage Safe and Effective Use of Opioid Therapy for Chronic, Noncancer Pain by Primary Care Providers. *Pain medicine (Malden, Mass)*. 2010;11(4):575-585. doi:10.1111/j.1526-4637.2010.00818.x

46. Trafton JA, Martins SB, Michel MC, et al. Designing an automated clinical decision support system to match clinical practice guidelines for opioid therapy for chronic pain. Report. *Implementation Science*. 2010;5:26. doi:10.1186/1748-5908-5-26

47. van der Meer HA, Doomen A, Visscher CM, Engelbert RHH, Nijhuis-van der Sanden MWG, Speksnijder CM. The additional value of e-Health for patients with a temporomandibular disorder: a mixed methods study on the perspectives of orofacial physical therapists and patients. *Disability and rehabilitation: Assistive technology*. 2022;1-13. doi:10.1080/17483107.2022.2094000

48. Verma SK, Chun S, Liu BJ. A web-based neurological pain classifier tool utilizing Bayesian decision theory for pain classification in spinal cord injury patients. SPIE; 2014:90390E-90390E-8.

49. Webers C, Beckers E, Boonen A, et al. Development, usability and acceptability of an integrated eHealth system for spondyloarthritis in the Netherlands (SpA-Net). *Rheumatic & musculoskeletal diseases open*. 2019;5(1):e000860-e000860. doi:10.1136/rmdopen-2018-000860

50. Yen PY, Lara B, Lopetegui M, et al. Usability and workflow evaluation of “RhEumAtic disease activity” (READY): A mobile application for rheumatology patients and providers. *Applied clinical informatics*. 2016;7(4):1007-1024. doi:10.4338/ACI-2016-03-RA-0036

51. Yin Z, Zhou L, He M, Chen X. MyHeadache: an intelligent headache diary mobile application to enhance patient compliance. IEEE; 2021:1-8.

52. Zheng H, Tulu B, Choi W, Franklin P. Using mHealth App to Support Treatment Decision-Making for Knee Arthritis: Patient Perspective. *EGEMS (Washington, DC)*. 2017;5(2):7-7. doi:10.13063/2327-9214.1284
