# Supplementary material for: Professional-Facing Digital Health Technology for the Care of Patients With Chronic Pain: Scoping Review
Source: J Med Internet Res. 2025 May 14;27:e66457. doi: 10.2196/66457 (PMC12120369; doi:10.2196/66457)
Supplement: Multimedia Appendix 5 [file jmir_v27i1e66457_app5.docx]

## Multimedia Appendix 4: Table of DHT intervention characteristics

Table 1: Table describing the characteristics of the 44 DHT interventions included in the scoping review, divided by type of DHT (remote patient monitoring, clinical decision support, education, assessment/diagnosis and combination) (N=52)

| Name of intervention | User features | Mode of intervention delivery | Data collected by intervention | Connected patient-facing system? | Development | Framework of development |
| --- | --- | --- | --- | --- | --- | --- |
| Remote patient monitoring | | | | | | |
| SCM-PM (Anderson et al, 2016) [1] | Displays patient information in an opioid management dashboard to inform opioid treatment:   - Key metrics for managing patients receiving COT - N and % of patients receiving COT - N patients with signed opioid treatment agreement - N patients with UDT screen within past 6 months - N patients completed pain interference assessment questionnaire within past 3 months, at least 1 behavioural health visit in the past year - List of HCP’s individual COT patients and which patients were due for any of these items | ‘Digital dashboard’ | None | No | Not reported | Not reported |
| Pain ROADMAP (Ireland and Andrews, 2019 [2]; Andrews et al, 2022) [3] | Data from patient app and wearable technology are exported to professional portal, displaying:   - Visual record that links specific activities to significant increases in pain - Patient pain intensity and objective activity values (pain score for every minute of the day) - Overactivity periods highlighted (severe pain aggravation, high-physical activity periods, prolonged sedentary task engagement) - Coded daily activities – rest, productive, leisure/social activity - N of statistical parameters | Online/web portal | Patient data (medication, daily physical activities and pain intensity) | Yes | - Co-designed by a clinician with >10 years experience and software engineer - 4 chronic pain patients and 3 HCPs tested mock-up of app prior to pilot; feedback used to make minor modifications to app | Not reported |
| Adhera for Rheumatology (adapted) (Benavent et al, 2022) [4] | Information from the patient-facing app is displayed on a web interface for the HCP to review when necessary:   - ePROs (specifically related to RA or SpA) inc. disease activity, joint information etc. - Flagged incidents by the patient | Online/web portal | Patient ePROs inc. PGA; s-TJC; s-SJC; HAQ; VAS; BASDAI; ASAS-HI | Yes | - Focus groups of 5 people were held to identify the needs of patients with RA and SpA, which led to development of a protocol - Patients used digital solution and completed questionnaires - Adaptation of Adhera to RA and SpA with multidisciplinary team of HCPs | Not reported |
| SATIE-PR (Bernard et al, 2022 [5]; Pers et al, 2021 [6]) | Information from the patient-facing app is displayed in a professional application:   - PROs inc. disease activity, general health condition, quality of life etc. - Notifies HCP when the data fill rate is less than 75% from patient or upon report of high disease activity (this can determine whether a face-to-face meeting is required) - Access to communication channel to patient | Digital tablet application | Patient PROs inc. RAPID-3; auto-DAS 28; general health questions (fever, treatment changes, disease flares, pain intensity based on VAS) | Yes | Not reported | Not reported |
| Manage My Pain (Bhatia et al, 2021 [7]) | HCPs can access functions to remotely assess patient progress, assign questionnaires and highlight clinically relevant trends:   - Patient pain and function trends across a pre-defined time period from patient-facing app - Assign questionnaires (pain and related domains) to patients to complete - Downloadable report with summary information | Online/web portal | Patient PROs inc. GAD-7; PHQ-9; PDI; PCS; PGIC; patient opioid consumption | Yes | Not reported | Not reported |
| PainCAS (Butler et al, 2016 [8]; McCaffrey et al, 2018 [9]) | HCPs can rapidly identify, track and appropriately treat patients at risk of aberrant drug-related behaviour; displays comprehensive patient information:   - Patient demographic information - Pain assessment and quality of life evaluation - Opioid risk assessments - Detailed initial assessment of pain complaint, medical and family history and current status - Follow-up assessment on changes in pain, function and risk along the continuum of care - Downloadable PDF report for summary information | Computer application/software | Patient demographic information; patient pain intensity, activity interference, mood, medication use and side effects; SOAPPVR; COMM-VR | Yes | - Expert advisory panel assisted to carry out a review to identify the domains and information considered important for documentation and assessment of chronic pain patients - Items for assessment further evaluated by 36 multidisciplinary HCPs using concept mapping - Series of patient and HCP cognitive interviews and usability testing sessions | Not reported |
| mHealth enabled integrated care (Colomina et al, 2021 [10]) | HCPs can access information on web-based platform (available to whole care team) to coordinate multidisciplinary care:   - Patient profile – inc. demographic information - Patient body map - Communication channel to other HCPs in different settings and patient - Patient baseline characteristics, risk stratification based on clinical, environmental and social risk assessments - Notifies on new tasks and messages | Online/web portal | Patient pain/discomfort; patient N steps and physical activity | Yes | Co-design process through an ‘iterative patient-centred process involving patients and stakeholders across different health settings’ – to produce a model for integrated care and an eHealth platform | Not reported |
| CAARING clinical professional website (Cordero-Tous et al, 2022 [11]) | Web-page which HCPs can open inside or outside the hospital for remote monitoring of chronic pain patients with neurostimulators; functions include:   - Accessing patient demographic and clinical data (from patient-facing app) - Control panel to manage different colour-coded alerts - Red alert indicates that clinical care is required for surgical wound infection - Yellow alert indicates clinical consideration due to possible deterioration in patient clinical course - Blue alert to warn HCPs of generator depletion | Online/web portal | Patient demographic and clinical data; DN-4; VAS; ODI; Patient perceived satisfactory improvement scale; SF-36; NDI; PREMs; event reports | Yes | Developed with multidisciplinary group of HCPs in 5 phases   1. Approval of idea about implants for CP patients in clinical session 2. Group of experts 3. Adaptation of clinical and educational protocol 4. Adaptation of technological platform to the clinical protocol 5. Quality assessment   No iterative improvements | John Hopkin’s tool for development of clinical applications (to guide system development) |
| Digital Model (Ekman et al, 2020 [12]) | Physiotherapist can access web interface to follow patient progress:   - Displays information on patient progress (from patient-facing interface) - Access to communication with patient - Coordinate and adjust patient treatment | Online/web portal | Patient information on ‘progress’ inc. assessments on mobility, pain, and health-related quality of life (EQ5D-5L) | Yes | Developed ‘over a decade’. No further information. | Not reported |
| MORPH companion portal (Fanning et al, 2020 [13]) | The portal allows the HCP to view real-time data from the patient-facing app; functions include:   - Summary of real-time patient data from patient-facing app - Patient daily ‘timeline’ bars which display colour-coded patterns of movement across the day (inactive periods blue and active periods green) - Daily minutes patient spent moving, daily steps, daily breaks - Patient goals – can be tailored based on patient data - Dietary log for nutritionist to tailor patient goals | Online/web portal | Patient physical activity and nutritional information | Yes | Developed as a series of iterative studies with patients with chronic pain – two phases:   1. Series of N-of-1 studies to identify and address technical and usability issues 2. RCT pilot study to evaluate the potential efficacy of the intervention and identify additional usability and technical issues | Not reported |
| Mida Rheuma DocBoard app (Fedkov et al, 2022 [14]) | HCP can access data from patient-facing app (Mida-Rheuma) in a web app; displays:   - Patient data inc. medication adherence and side-effects - Physicians can add to patient data inc. joint count and laboratory test results - Patient disease scores - Patient disease management programs and action plans - Access to standardized questionnaires | Online/web portal | PROs inc. SF-36 total score; PTtGADA; PPAIN; RADAI-5; SDAI; CDAI; BASDAI; ASDAS; BMI; HAQ; BFI; PHQ-9 | Yes | ‘Developed in accordance with EULAR guidelines recommendations’. No further information. | Not reported |
| Geisinger’s HCP dashboard (Han et al, 2022 [15]) | HCP can access dashboard to monitor patients; displays patient-specific data from a patient-facing app and wearable health technology to be reviewed prior to physical visits:   - Patient pain intensity on daily basis, same time every day - Breakthrough pain – location and description - Patient sleep patterns - Patient treatments - Patient physical activity | Digital dashboard | Patient information on pain, physical activity and sleep patterns | Yes | Developed in response to HCP and patient usability testing. No further information. | Not reported |
| German xSpA live app (Kempin et al, 2022 [16]) | Data from patient-facing app can be exported onto a physician interface; displays graphical information on ePROs on pain, disease activity, physical function. | App ‘Interface’ | ePROs inc. BASDAI; patient information on pain, disease activity and physical function | Yes | Not reported | Not reported |
| eDOL app (Kerckhove et al, 2022 [17]) | Data from patient-facing app is displayed in a web interface for physicians:   - Graphical summaries of ePROs (e.g., pain, psychological functioning etc.) - Ergonomic dashboard for physicians to find patients with tabs including management, health measures and questionnaires - Management tab – all medical records - Health measures tab – graphic display of weekly assessments - Questionnaires tab – all questionnaires completed by patients - Medical forms for consultations – diagnosis, current treatments and examination results - Option to activate new questionnaires to be completed by patients on app - Diagnostic questionnaires | Online/web portal | PROs inc. general questionnaires on sociodemographic, lifestyle and professional data; Pain Beliefs and Perceptions Inventory; Evaluation of level of precariousness; Injustice Experience Questionnaire; Maslach Burn-out Inventory; Toronto Alexithymia Scale; Life Orientation Test-Revised; Belief in a just world; Job Content Questionnaire; Big Five Inventory and questionnaires, assessing symptoms, comorbidities, and psychological and physiological states related to chronic pain, Brief Pain Inventory; Medical Outcomes Study Sleep Scale; Tampa Scale of Kinesiophobia; Pain Catastrophizing Scale; Fear-avoidance beliefs; EQ-5D-3L; Hospital Anxiety Depression Scale; Satisfaction With Life Scale; Subjective Cognitive Complaints; Neuropathic Pain Scale Inventory; Western Ontario and McMaster Universities; Rheumatoid Arthritis Impact of Disease; Roland Morris Disability Questionnaire; Irritable Bowel Severity Scoring System; Fibromyalgia Impact Questionnaire; Headache Impact Test | Yes | Not reported | Not reported |
| eCoach Pain (Lamper et al, 2021 [18]) | HCPs can access a secure webpage from their own device to monitor patient treatment progress based on data from patient-facing app; features include:   - Displays information on real-time patient clinical data (biopsychosocial complaints) from PROs (graphs and overview) - Access to patient pain diary - Access to communication with patients (chat function) - Flag/alert system for treatment risk (red for high risk, orange for medium risk and green for low risk) | Online/web portal | PROs inc. STarT MSK tool (activity level, anxiety, depression, thoughts about CMP); VAS; questions about biopsychosocial complaints and patient background | Yes | Developed iteratively in collaboration with multiple stakeholders including researchers, technical experts, patients and HCPs. No further information. | Not reported |
| System of disease management application (no name) (Li et al, 2023 [19]) | HCPs can access the rheumatologist interface to monitor patients based on data input in patient-facing app; displays:   - PROs – patient disease activity - Patient demographic data and clinical history - Patient laboratory results, medications and perceived adverse reactions - Alert flag function which showed colour-coded flags for HCP attention   Red flag indicated disease activity exacerbation or worsening assessment results | ‘Interface’ | PROs inc. DAS28-CRP; patient demographic and clinical information/history; lab results | Yes | Not reported | Not reported |
| Reumanet Bernhoven (Muskens et al, 2021 [20]) | Platform is available to rheumatologists and other HCPs (chwith permission from patient) and displays:   - PROs inc. pain, disease activity, functional capacity, health-related quality of life - Patient information on education status, working status, smoking status, etc. - Access to communication with patient (chat function) | ‘Platform’ | PROs inc. HAQ; SF-36; RAID; VAS | Yes | Not reported | Not reported |
| Web services pain diary (no name) (Pombo et al, 2012 [21]) | HCPs can monitor patient’s diary/symptoms and communicate with patients:   - HCPs can determine the frequency of patient recording in patient-facing diary - Displays patient’s symptoms and other diary inputs; notifies the HCP when data is available or not available - Access to two-way communication with patient | Online/web portal | Patient diary data | Yes | Not reported | Not reported |
| Limbr Suite (Selter et al, 2018 [21]) | HCPs can access the Limbr Suite to remotely monitor patients and communicate; displays information on:   - PROs inc. patient pain, functional ability, mood, physical activity (WHT) - Patient current treatment - HCPs can also access communication channel to patient (Limbr chat app) - HCPs can send reminders to patients to complete PROs | Not specified | PROs inc. Your Activities of Daily Living; Medications of Daily Living; Photographic Affect Meter; patient activity levels monitored on WHT | Yes | Not reported | Not reported |
| SpA-Net (Webers et al, 2019 [23]) | HCPs can access system for comprehensive disease management of spondylarthritis patients, using data from patient-facing app; displays:   - 3 tabs: dashboard; visit; and data input and reporting - Dashboard tab - Patient personal information inc. past presence of symptoms, current medication use, summary of recent visits, patient notes and graphical summaries of disease scores, health questionnaire results - Disease activity graph is colour-coded - Visit tab – HCPs can enter new outpatient visit and includes selection of items for clinical record-keeping - Adverse events can be reported - Data input and reporting tab – all items of SpA-Net - Additional dashboard for HCPs to access aggregated data on clinical indicators for quality improvement, comparing to other centres | Digital tablet application | PROs inc. ASDAS; HAQ-S; SF-36; EMR information | Yes | Developed in iterative process of 4 phases with various stakeholders including patients and HCPs:   1. Content and design 2. Technical development of database and EMR 3. Internal and external testing 4. Implementation | Not reported – unclear where 4 phases came from |
| READY (Yen et al, 2016 [24]) | Shared platform integrating data from patients and HCPs to monitor arthritic disease activity, information from patient-facing app is displayed in professional application:   - PROs inc. pain, quality of life, tender joint count etc. - Graphical representations of patient information - HCPs can document patient diagnosis and medical treatment - Prompted instructions and error messages indicate for HCPs to help with navigation | Digital tablet application | PROs inc. MDHAQ; VAS; Fatigue VAS; Patient Global VAS; SF-12; EQ5D; RADAI; PASS; Tender joint count; swollen joint count; Physician Global VAS; disease characteristics | Yes | Not reported | Not reported |
| Monitoring system (no name) (Zheng et al, 2017 [25]) | Monitoring system for advanced knee arthritis patients with daily reports of pain (and ‘other symptoms’ from patient-facing app, which can be used to inform HCP treatment decisions. Limited information on user features. | Mobile application | Patient information (pain, arthritis symptoms, ‘good’ and ‘bad’ days) | Yes | System developed using user-centred design principles of interviewing and conducting focus groups with patients and HCPs. No further information. | User-centred design principles |
| Clinical decision support | | | | | | |
| Chronic pain OneSheet (Apathy et al, 2022 [26]) | HCPs can access functions in order to complete guideline-recommended opioid mitigation:   - Ordering and reviewing urine toxicology results - Reviewing prescription drug monitoring program reports - Ordering naloxone - Verifying an opioid treatment agreement - Reviewing current medications for opioid dose and concurrent opioid benzodiazepine prescribing   Displays patient information on:   - Pain-related diagnoses – any visit diagnoses the patient has historically reported/IC-10 diagnosis codes - Opioid (agonist or antagonist) Rx or benzodiazepine Rx – current medications - Opioid prescription morphine equivalent daily dose - Quick orders - Appointment history - e-consents - PDMP link - Pain, enjoyment, and general activity (PEG) scale – 3-item - Patient goals - Urinary drug screening and drug confirmation results - Treatment tracker | Unspecified | Patient data can be input into the tool – inc. medications, documentation, pain etc. | No | ‘User-centred’ studies – 89 cognitive interviews and visit observations with 20 HCPs and other stakeholders  Reported in previous studies Militello et al (2020) and Harle et al (2018) | Not reported – could be in previous studies |
| CDSS Back-Up system (Jansen-Kosterink et al, 2021 [27]) | Limited information. Example case scenarios of chronic pain patients can be used to help with complex clinical decision support, specifically tailored to different professions inc. GPs, primary care physiotherapists and other HCPs. | Online/web portal | None | No | Not reported | Not reported |
| Movement is Life Shared Decision Making Tool (Johnson et al, 2021 [28]) | Tool produces a predictive output page based on patient data to help decision making on treatment pathways and provide a framework for patient-centred discussions; functions include:   - Displays patient clinical and demographic information in personalised output page - Displays information on patient pain from PROMS - Displays information on patient physical activity from PROMS - Access to and guidance on treatment options (evidence-based) - Displays alternative treatments - Displays predicted impact of treatment on patient outcomes (pain, activity, economic productivity) - Compares impact of possible treatment to no treatment on patient outcomes - Access to best practice treatment pathways | Computer/web application | Patient demographic data | No | Multidisciplinary stakeholders led the development of the tool. No further information. | Not reported |
| Rheuma Care Manager and flare prediction tool (Labinsky et al, 2023 [29]) | The RCM has two parts: (1) patient overview; (2) flare risk prediction tool (AI-powered):   - Patient overview – displays patient clinical history (e.g., previous and current medication, demographic data etc.) and a visual timeline of disease activity and medication - Flare risk prediction tool – displays the predicted risk of disease flare for patient (bar graphs for two scenarios regarding medication) | Not specified | None | No | Flare risk prediction tool machine learning model was developed based on data from clinical routines with 50 RA patients. No further information on RCM. | Not reported |
| CDS for opioid management of chronic non-cancer pain (Price-Haywood et a 2018 [30]; 2020 [31]) | HCPs can access opioid risk tool and other functions to support treatment decisions for patients with chronic non-cancer pain; features include:   - Opioid risk tool for when prescribing opioids, if not already documented and up-to-date according to patient ae - Alert system showing flags of high risk patients for opioids - Health maintenance tool – displays whether patients are up to date on chronic opioid management best practices - Access to documentation including pain agreement - Recommended functional assessments - Access to other clinical questionnaires e.g., for psychiatric symptoms - Hyperlinks to resources such as Louisiana Board of Pharmacy Prescription Monitoring Program data and pain management agreement - Epic banner – to alert HCPs of medium and high risk patients with quick link to health maintenance tool - Chronic opioid management guide with links to resources e.g., guideline fact sheet, urine drug screening etc. | Computer application | PEG 3-item scale; PHQ-4; PHQ-914; GAD-7; BPI | No | Not reported | Not reported |
| ATHENA-OT (Trafton et al 2010 [32]; 2010 [33]) | HCPs can access system to support decisions on safe and effective use of opioid therapy for chronic non-cancer pain; features include:   - Displays information on patient clinical data (summary, assessments, orders) - Displays information on patient current treatment - Access to documentation (agreements) - Guidance/support on treatment decisions (opioid) | Computer application | None | No | System was developed through iterative evaluation of usability in 2 phases with HCPs (simulation and clinic based testing) to identify changes to the interface:   1. Before major redesign – plan for revision made 2. After major redesign – additional revisions | Used ADDIE (analysis, design, development, implementation and evaluation) to guide system development |
| Pain Management Advisor (Knab et al, 2001 [34]) | CDS system can generate specific treatment recommendations for physicians in the management of chronic pain patients.   - Recommendations are provided in a prioritised list of therapeutic options - HCP can access explanations, therapeutic rationale and therapy guidelines - HCP can access next level of therapy (if not satisfied with primary recommendation) | Computer application | Patient clinical information (to feed into CDS algorithm) | No | Not reported | None |
| Education | | | | | | |
| TREK ‘My Knee’ Toolkit (Goff et al, 2023 [35]) | HCPs had access to a ‘for clinicians’ tab which included features to help improve the communication between HCP and patient:   - Introductory video - Quick access links – guides to help improve communication - Resources section for HCPs to find and share infographics or resources | Online/web portal | None | Yes | Co-designed with patients and HCPs in 3 workshops using iterative process  Three stages:   1. Understand and define 2. Prototype 3. Test and iterate | Good Things Foundation pathfinder model for digital health inclusion (2019) (to guide system development) |
| Rheumality (Klemm et al, 2021 [36]) | VR app to educate HCPs and medical students on inflammatory arthritis:   - Imaging data on arthritic joints and bone structures - Case scenarios of RA patients with clinical information disease history - HCP can indicate to the typical arthritic joint and bone pathologies on images - Tests for HCPs to estimate how much patient is restricted in different areas of life based on images | VR application | HCP user performance | No | Based on anonymized patient cases with corresponding clinical and imaging data | Not reported |
| The chronic pain and headache management TeleECHO clinic (Shelley et al, 2017 [37]; Katzman et al, 2013 [38]) | Digital ECHO pain curriculum provides HCPs with education on chronic pain patient care via a professional-facing website:   - ‘Hub-centred’ – topics covered in curriculum displayed with schedules - Topics include pain management from IASP curriculum - HCPs can access advanced modules on primary care for chronic pain - Topics include: chronic pain, clinical interviewing, interpreting radiology results, formulating interdisciplinary treatment plan, traumatic brain injury, sleep medicine, the spine and CRPS - HCPs can also access case presentations | Online/web portal | None | No | Not reported | Not reported |
| Assessment and diagnosis | | | | | | |
| SymptomMapper (Neubert et al, 2018 [39]) | The software app had a doctor interface to facilitate assessment diagnosis; features included:   - Patient body map and symptom specification - Symptom specification module – to enter common diagnostic findings in a bodily examination - Access to pain assessments (e.g., VAS) | Digital tablet application | Patient pain information (body map and VAS) | Yes | Two studies on development with patients and doctors:   1. Usability – questionnaires and user observation leading to user interface modifications 2. Improved version of app retested in chronic pain patients | Partially followed design guidelines from a previous study which developed a computerised pain assessment tool (not framework) |
| Demetra app (Papageorgiou et al, 2021 [40]) | Webserver for HCPs to facilitate clinical genomic diagnosis of endometriosis; features include:   - Upload patient genetic data - Analyse patient genetic data to identify variants relating to endometriosis - Diagnosis of endometriosis - Access to links to online databases - Access to patient clinical information (genetic data) - Educational resources for diagnosis - Displays patient’s clinical information (genetic data) in chart bar form | Computer application | Patient genetic information | No | System development not reported. Gene datasets used in app identified through literature review. | Not reported |
| Combination (remote patient monitoring, CDS, education, assessment and diagnosis) | | | | | | |
| Chronic pain treatment tracker (Allen et al 2022 [41]; Harle et al, 2019 [42]) | Tool for remote patient monitoring and CDS. HCPs can use the Chronic Pain Treatment Tracker to help inform decisions on treatment; features include:   - Displays information on patient current treatment (split into 6 categories including oral medications, topical medications, referrals, interventions, integrative medicine, lifestyle changes) - Access to and guidance on future treatment options for patient - Edit patient clinical notes - Access to other clinical notes (from other professionals) - Displays information on patient past treatments (inc. Reason for discontinuation) - Access to caution list (high risk treatments) - Access to educational resources on treatment types - Access to referrals for treatment - Access to link for medication orders | Computer application | None | No | - Iteratively designed to support 4 decision requirements - Prototypes developed through user testing (clinician interviews and design workshop) - Based on a series of studies (Harle et al, 2018; Diulio et al, 2020; Militello et al, 2020) | ‘Decision-centred design’ |
| PMSS-PC (Dhingra et al, 2021 [43]) | Tool for CDS and education of PCPs; functions include:   - Alerts for patients with clinically significant pain from PROMs - Displays information on patient pain, physical and psychosocial functioning - Access to links to content that covered pain assessment - Access to links to content on the use of primary therapy with analgesic intent - Access to guidelines (drug and non-drug treatment for musculoskeletal and neuropathic pain) - Recommended order sets (prescribing of analgesic drugs and other treatments) - Access to referral links for non-pharmacologic pain therapies such as acupuncture, psychotherapy, and physical therapy - Option to access the new PMSS-PC content from the alert - Pain assessment tool - Templated tool for recording pain characteristics. - Access to links to other questionnaires (the Patient Health Questionnaire, the Drug Abuse Screening Test, the Alcohol Use Disorders Identification, the Opioid Risk Tool, and the Pain Assessment and Documentation Tool) - Access to 6 webinars for education (topics included: managing high-risk chronic pain patients, optimizing non-opioid pharmacotherapy, integrative medicine approaches, exit strategies for opioid pharmacotherapy, and management of psychiatric comorbidities) | Online/web portal | Patient clinical information; results to following questionnaires: Patient Health Questionnaire, the Drug Abuse Screening Test, the Alcohol Use Disorders Identification, the Opioid Risk Tool and the Pain Assessment and Documentation Tool | No | Developed by pain specialists and technical specialists from the Institute for Family Health. No further information. | Not reported |
| CDSS for primary headache diagnosis (no name) (Dong et al, 2014 [44]) | CDS system can make computerised diagnoses of chronic pain (e.g., chronic migraine, persistent headache, chronic tension-type headache) based on clinical patient information input by physician. | Computer application | Patient clinical information on headache disorders (location, duration, attack frequency, severity, accompanying symptoms, aura) | No | - Knowledge base for CDSS developed by headache specialists using guidelines - Validated in prospective study with 543 headache patients | None |
| Online TMS for chronic low back pain (Kampusch et al, 2022 [45]) | Tool for remote patient monitoring and CDS - HCP can access a web-based patient management system which displays information from a patient-facing app to monitor patients and support treatment decisions:   - Displays information on patient pain from PROMS and wearable - Displays information on patient current treatment - Displays information on patient heart rate and blood pressure from wearable - Displays information patient wellbeing from PROMS - Track patient treatment outcomes and progress | Online/web portal/application | Patient information on pain, subjective wellbeing, pain medication, blood pressure, heart rate | Yes | Not reported | Not reported |
| MyHeadache (Yin et al, 2021 [46]) | Tool for diagnosis and CDS – professional components include a headache reporting module, headache diagnosis and pain relief module (CDS) and doctor-patient communication module:   - Headache reporting module – weekly, monthly, yearly reports based on data from patient-facing diary module to identify trends of headache attacks and clinical history - CDSS module – AI diagnosis function using ICHD-3 to diagnose chronic pain types - Communication module – can be used by physician to converse and share details with patients | Online/web portal | Patient information in diary inc. headache frequency, onset times, duration, location of pain, nature of pain, medication etc. | Yes | Not reported | Not reported |
| Tool for CDS and diagnosis (no name) (Peiris et al, 2-14 [47]) | Tool for CDS and diagnosis – which can be used by HCPs to diagnose chronic pain; features include:   - Input patient information on pathology - Access to clinical assessment - Diagnosis of back pain type - Displays patient clinical information - Access to treatment considerations | Computer ‘Interface’ | Patient clinical information (pain, pain duration, any features suggesting sciatica/radiculopathy, features suggesting spinal stenosis, etc.) | No | - Decision support algorithm developed through literature review and validation study with 16 hypothetical cases compared to expert clinical decisions - Panel of 4 experts reviewed prototype - User interface designed through technical user acceptance testing with GPs | Not reported |
| Education eHealth tool for SCS (Thomson et al 2020 [48]; 2021 [49]) | Tool for CDS and education – to support and educate HCPs specifically on referral of chronic pain patients to spinal chord stimulation:   - Patient profile – information on previous spinal surgery, main location of pain, dominant pain type, response to other treatments - CDS - Panel recommendation ranging from strongly recommended, recommended to not recommended for referral to SCS - Displays patient information regarding psychosocial factors | Not specified | Patient clinical and psychosocial information | No | CDS panel recommendation algorithm was based on several panel meetings with multidisciplinary HCPs and literature review. System development not reported. | Not reported |
| Physitrack (van der Meer, 2022 [50]) | Tool for treatment and remote patient monitoring - Online platform for physiotherapists to remotely monitor patients and deliver treatment (limited information on user features reported); features include:   - Access to communication with patient (chat function) - Access to online consultations with patients - Access to clinical assessments - Access to treatment programs (exercise)   Physiotherapists can decide which features patients can user and how to fit Physitrack into their therapy programs | Online/web portal | None | Yes | Not reported | Not reported |
| Web-based pain classifier system (no name) (Verma et al, 2014 [51]) | Tool for CDS and diagnosis – system can be used by HCPs to classify pain types caused by spinal cord injury to diagnose chronic pain:   - HCPs can mark locations specifying pain locations on a body map - Body map – various grid locations which can be highlighted by the HCP - Pain classification questionnaires - Results of pain classification questionnaire generated by Bayesian algorithm which indicates % probability of a pain to be a certain type | Computer application | Patient pain locations and other pain information | No | Retrospective pilot study to evaluate the initial prototype conducted. Algorithm for CDS developed using Bayesian decision theory. No further information on development of system. | Not reported |
| Web-based diagnosis and CDS system (no name) (Lin et al, 2006 [52]) | Tool for CDS and diagnosis – web-based system to support HCPs to diagnose chronic low back pain; two interfaces:   - Diagnosis interface – HCP (or patient) can input patient symptoms which generates diagnosis - HCP can override diagnostic decision - HCP can access explanatory panel to review CDS - Displays information on patient pain | Online/web portal | HCP (or patient) can input patient information on pain | No | ‘system validation based on a modified Turing test’ | Not reported |

*Table* 2*: The most common user features of the 44 DHT in each categorical theme produced by the inductive content analysis*

| **Categorical theme** | **Top 3 most common user features** |
| --- | --- |
| Theme 1: Guiding initial consultation | - Access to patient medical background and history (N=15) - View of patient’s current treatments (N=12) - Direct access to relevant clinical assessments (N=9) |
| Theme 2: Supporting chronic pain management | - Treatment decision support (generation of future treatment pathways) (N=9) - Ability to prescribe pharmacological treatment (N=3) - Direct access to treatment programs (N=1)   *Sub-theme: managing opioid intervention and risk*   - Access to patient’s current opioid treatment (N=9) - Displays opioid risk information (N=2) - Direct access to relevant consent documentation (N=2) |
| Theme 3: Facilitating ongoing patient management | - Access to patient pain assessment results (no connected patient-facing DHT) (N=6) - Access to patient physical activity assessment results (no connected patient-facing DHT) (N=3) - Access to patient psychosocial and anxiety assessment results (no connected patient-facing DHT) (N=2)   *Sub-theme: supporting self-management*   - Displays pain PROs (N=19) - Access to a direct communication channel to patient e.g., chat function (N=9) - Displays patient’s physical activity data e.g., for WHT (N=7) |
| Theme 4: Supporting routine HCP duties | - Access to HCP educational content (N=7) - Access to direct resource links (N=5) - Ability to make clinical notes (N=4) |

^a^ PROs = patient reported outcomes; WHT = wearable health technology; HCP = health and care professional

### References

1. Anderson DR, Zlateva I, Coman EN, Khatri K, Tian T, Kerns RD. Improving pain care through implementation of the Stepped Care Model at a multisite community health center. Report. *Journal of Pain Research*. 2016;9:1021. doi:10.2147/JPR.S117885
2. Ireland D, Andrews N. Pain ROADMAP: A mobile platform to support activity pacing for chronic pain. In: Ebooks IP, ed. *Digital health: changing the way healthcare is conceptualised and delivered*. 2019:89-94.
3. Andrews NE, Ireland D, Deen M, Varnfield M. Clinical utility of a mHealth assisted intervention for activity modulation in chronic pain: The pilot implementation of pain ROADMAP. *European Journal of Pain*. 2023;27(6):749-765. doi:10.1002/ejp.2104
4. Benavent D, Fernández-Luque L, Núñez-Benjumea FJ, et al. Monitoring chronic inflammatory musculoskeletal diseases mixing virtual and face-to-face assessments—Results of the digireuma study. *PLOS Digital Health*. 2022;1(12):e0000157. doi:10.1371/journal.pdig.0000157
5. Bernard L, Valsecchi V, Mura T, et al. Management of patients with rheumatoid arthritis by telemedicine: connected monitoring. A randomized controlled trial. *Joint Bone Spine*. 2022;89(5):105368. doi:10.1016/j.jbspin.2022.105368
6. Pers Y-M, Valsecchi V, Mura T, et al. A randomized prospective open-label controlled trial comparing the performance of a connected monitoring interface versus physical routine monitoring in patients with rheumatoid arthritis. *Rheumatology*. 2021;60(4):1659-1668. doi:10.1093/rheumatology/keaa462
7. Bhatia A, Jamal K, Janmohamed T, et al. User Engagement and Clinical Impact of the Manage My Pain App in Patients With Chronic Pain: A Real-World, Multi-site Trial. *JMIR mHealth and uHealth*. 2021;9(3)doi:10.2196/26528
8. Butler SF, Zacharoff KL, Charity S, et al. Impact of an Electronic Pain and Opioid Risk Assessment Program: Are There Improvements in Patient Encounters and Clinic Notes? Article. *Pain Medicine*. 2016;17:2047+. doi:10.1093/pm/pnw033
9. McCaffrey SA, Black RA, Butler SF. Psychometric evaluation of the PainCAS Interference with Daily Activities, Psychological/Emotional Distress, and Pain scales. *Quality of life research*. 2018;27(3):835-843. doi:10.1007/s11136-017-1766-3
10. Colomina J, Reis D, Torra M, et al. Implementing mHealth-Enabled Integrated Care for Complex Chronic Patients With Osteoarthritis Undergoing Primary Hip or Knee Arthroplasty: Prospective, Two-Arm, Parallel Trial. *Journal of Medical Internet Research*. 2021;doi:10.2196/28320
11. Cordero Tous N, Santos Martín L, Sánchez Corral C, et al. Development of an integrated solution for patients with neurostimulator for chronic pain in times of COVID-19: A mobile application with a support center. *Neurocirugía (English Edition)*. 2022;33(6):318-327. doi:10.1016/j.neucie.2021.12.001
12. Ekman B, Nero H, Lohmander LS, Dahlberg LE. Costing analysis of a digital first-line treatment platform for patients with knee and hip osteoarthritis in Sweden. Report. *PLoS ONE*. 2020;15:e0236342. doi:10.1371/journal.pone.0236342
13. Fanning J, Brooks AK, Ip E, et al. A Mobile Health Behavior Intervention to Reduce Pain and Improve Health in Older Adults With Obesity and Chronic Pain: The MORPH Pilot Trial. Clinical Trial. *Frontiers in Digital Health*. 2020;2doi:10.3389/fdgth.2020.598456
14. Fedkov D, Berghofen A, Weiss C, et al. Efficacy and safety of a mobile app intervention in patients with inflammatory arthritis: a prospective pilot study. *Rheumatology International*. 2022;42(12):2177-2190. doi:10.1007/s00296-022-05175-4
15. Han JJ, Graham JH, Snyder DI, Alfieri T. Long-term Use of Wearable Health Technology by Chronic Pain Patients. *The Clinical journal of pain*. 2022;38(12):701-710. doi:10.1097/AJP.0000000000001076
16. Kempin R, Richter JG, Schlegel A, et al. Monitoring of Disease Activity With a Smartphone App in Routine Clinical Care in Patients With Axial Spondyloarthritis. *Journal of rheumatology*. 2022;49(8):878-884. doi:10.3899/jrheum.211116
17. Kerckhove N, Delage N, Cambier S, et al. eDOL mHealth App and Web Platform for Self-monitoring and Medical Follow-up of Patients With Chronic Pain: Observational Feasibility Study. *JMIR formative research*. 2022;6(3):e30052-e30052. doi:10.2196/30052
18. Lamper C, Huijnen I, Mooij Md, Köke A, Verbunt J, Kroese M. An ecoach-pain for patients with chronic musculoskeletal pain in interdisciplinary primary care: A feasibility study. *International journal of environmental research and public health*. 2021;18(21):11661. doi:10.3390/ijerph182111661
19. Li C, Huang J, Wu H, et al. Management of Rheumatoid Arthritis with a Digital Health Application: A Multicenter, Pragmatic Randomized Clinical Trial. *JAMA network open*. 2023;6(4):E238343-e238343. doi:10.1001/jamanetworkopen.2023.8343
20. Müskens WD, Rongen-van Dartel SAA, Vogel C, Huis A, Adang EMM, van Riel PLCM. Telemedicine in the management of rheumatoid arthritis: maintaining disease control with less health-care utilization. *Rheumatology advances in practice*. 2021;5(1):rkaa079-rkaa079. doi:10.1093/rap/rkaa079
21. Pombo N, Araújo P, Viana J, Junior B, Serrano R. Contribution of web services to improve pain diaries experience. Int Assoc Engineers-Iaeng; 2012; 589-592.
22. Selter A, Tsangouri C, Ali SB, et al. An mHealth app for self-management of chronic lower back pain (Limbr): Pilot study. *JMIR mHealth and uHealth*. 2018;20(9):e179-e179. doi:10.2196/mhealth.8256
23. Webers C, Beckers E, Boonen A, et al. Development, usability and acceptability of an integrated eHealth system for spondyloarthritis in the Netherlands (SpA-Net). *Rheumatic & musculoskeletal diseases open*. 2019;5(1):e000860-e000860. doi:10.1136/rmdopen-2018-000860
24. Yen PY, Lara B, Lopetegui M, et al. Usability and workflow evaluation of “RhEumAtic disease activity” (READY): A mobile application for rheumatology patients and providers. *Applied clinical informatics*. 2016;7(4):1007-1024. doi:10.4338/ACI-2016-03-RA-0036
25. Zheng H, Tulu B, Choi W, Franklin P. Using mHealth App to Support Treatment Decision-Making for Knee Arthritis: Patient Perspective. *EGEMS (Washington, DC)*. 2017;5(2):7-7. doi:10.13063/2327-9214.1284
26. Apathy NC, Sanner L, Adams MCB, et al. Assessing the use of a clinical decision support tool for pain management in primary care. *JAMIA Open*. 2022;5(3):ooac074. doi:10.1093/jamiaopen/ooac074
27. Jansen-Kosterink S, van Velsen L, Cabrita M. Clinician acceptance of complex clinical decision support systems for treatment allocation of patients with chronic low back pain. *BMC medical informatics and decision making*. 2021;21(1):137-137. doi:10.1186/s12911-021-01502-0
28. Johnson CB. A Personalized Shared Decision-Making Tool for Osteoarthritis Management of the Knee. *Orthopaedic Nursing*. 2021;40(2)doi:10.1097/NOR.0000000000000739
29. Labinsky H, Ukalovic D, Hartmann F, et al. An AI-Powered Clinical Decision Support System to Predict Flares in Rheumatoid Arthritis: A Pilot Study. *Diagnostics (Basel)*. 2023;13(1):148. doi:10.3390/diagnostics13010148
30. Price-Haywood EG, Robinson W, Harden-Barrios J, Burton J, Burstain T. Intelligent clinical decision support to improve safe opioid management of chronic noncancer pain in primary care. *The Ochsner journal*. 2018;18(1):30-35. doi:10.1043/TOJ-17-0093
31. Price-Haywood EG, Burton J, Burstain T, et al. Clinical Effectiveness of Decision Support for Prescribing Opioids for Chronic Noncancer Pain: A Prospective Cohort Study. *Value in health*. 2020;23(2):157-163. doi:10.1016/j.jval.2019.09.2748
32. Trafton J, Martins S, Michel M, et al. Evaluation of the Acceptability and Usability of a Decision Support System to Encourage Safe and Effective Use of Opioid Therapy for Chronic, Noncancer Pain by Primary Care Providers. *Pain medicine (Malden, Mass)*. 2010;11(4):575-585. doi:10.1111/j.1526-4637.2010.00818.x
33. Trafton JA, Martins SB, Michel MC, et al. Designing an automated clinical decision support system to match clinical practice guidelines for opioid therapy for chronic pain. Report. *Implementation Science*. 2010;5:26. doi:10.1186/1748-5908-5-26
34. Knab JH, Wallace MS, Wagner RL, Tsoukatos J, Weinger MB. The Use of a Computer-Based Decision Support System Facilitates Primary Care Physicians’ Management of Chronic Pain. *Anesthesia & Analgesia*. 2001;93(3):712-720. doi:10.1097/00000539-200109000-00035
35. Goff AJ, De Oliveira Silva D, Ezzat AM, Crossley KM, Pazzinatto MF, Barton CJ. Co-design of the web-based ‘My Knee’ education and self-management toolkit for people with knee osteoarthritis. *Digital Health*. 2023;9doi:10.1177/20552076231163810
36. Klemm P, Kleyer A, Tascilar K, et al. A virtual reality based app to educate health care professionals and medical students about inflammatory arthritis: Feasibility study. *JMIR serious games*. 2021;9(2):e23835-e23835. doi:10.2196/23835
37. Shelley BM, Katzman JG, Comerci GD, et al. ECHO pain curriculum: Balancing mandated continuing education with the needs of rural health care practitioners. *The Journal of continuing education in the health professions*. 2017;37(3):190-194. doi:10.1097/CEH.0000000000000165
38. Katzman JG. Making Connections: Using TeleHealth to Improve the Diagnosis and Treatment of Complex Regional Pain Syndrome, an Underrecognized Neuroinflammatory Disorder. *Journal of Neuroimmune Pharmacology*. 2013;8(3):489-493. doi:10.1007/s11481-012-9408-6
39. Neubert TA, Dusch M, Karst M, Beissner F. Designing a tablet-based software app for mapping bodily symptoms: Usability evaluation and reproducibility analysis. *JMIR mHealth and uHealth*. 2018;6(5):e127-e127. doi:10.2196/mhealth.8409
40. Papageorgiou L, Zervou MI, Vlachakis D, et al. Demetra Application: An integrated genotype analysis web server for clinical genomics in endometriosis. *International journal of molecular medicine*. 2021;47(6):1. doi:10.3892/ijmm.2021.4948
41. Allen KS, Danielson, E. C., Downs, S. M., Mazurenko, O., Diiulio, J., Salloum, R. G., ... & Harle, C. A. . Evaluating a Prototype Clinical Decision Support Tool for Chronic Pain Treatment in Primary Care. *Applied Clinical Informatics* 2022;13(3):602-611. doi:10.1055/s-0042-1749332
42. Harle CA, DiIulio J, Downs SM, et al. Decision-Centered Design of Patient Information Visualizations to Support Chronic Pain Care. *Applied clinical informatics*. 2019;10(4):719-728. doi:10.1055/s-0039-1696668
43. Dhingra L, Schiller R, Teets R, et al. Pain Management in Primary Care: A Randomized Controlled Trial of a Computerized Decision Support Tool. *The American Journal of Medicine*. 2021;134(12):1546-1554. doi:10.1016/j.amjmed.2021.07.014
44. Dong Z, Yin Z, He M, Chen X, Lv X, Yu S. Validation of a guideline-based decision support system for the diagnosis of primary headache disorders based on ICHD-3 beta. *The journal of headache and pain*. 2014;15:40. doi:10.1186/1129-2377-15-40
45. Kampusch S, Edegger K, Mayr P, et al. Integrated Platform for the Management of Chronic Low Back Pain. In: Press I, ed. *dHealth*. 2022:260-261.
46. Yin Z, Zhou L, He M, Chen X. MyHeadache: an intelligent headache diary mobile application to enhance patient compliance. IEEE; 2021:1-8.
47. Peiris D, Williams C, Holbrook R, et al. A web-based clinical decision support tool for primary health care management of back pain: Development and mixed methods evaluation. *JMIR research protocols*. 2014;3(2):e17-e17. doi:10.2196/resprot.3071
48. Thomson S, Huygen F, Prangnell S, et al. Appropriate referral and selection of patients with chronic pain for spinal cord stimulation: European consensus recommendations and e‐health tool. *European journal of pain*. 2020;24(6):1169-1181. doi:10.1002/ejp.1562
49. Thomson S, Huygen F, Prangnell S, et al. Applicability and Validity of an e-Health Tool for the Appropriate Referral and Selection of Patients With Chronic Pain for Spinal Cord Stimulation: Results From a European Retrospective Study. *Neuromodulation: Technology at the Neural Interface*. 2023;26(1):164-171. doi:10.1016/j.neurom.2021.12.006
50. van der Meer HA, Doomen A, Visscher CM, Engelbert RHH, Nijhuis-van der Sanden MWG, Speksnijder CM. The additional value of e-Health for patients with a temporomandibular disorder: a mixed methods study on the perspectives of orofacial physical therapists and patients. *Disability and rehabilitation: Assistive technology*. 2022;1-13. doi:10.1080/17483107.2022.2094000
51. Verma SK, Chun S, Liu BJ. A web-based neurological pain classifier tool utilizing Bayesian decision theory for pain classification in spinal cord injury patients. SPIE; 2014:90390E-90390E-8.
52. Lin L, Hu PJ-H, Liu Sheng OR. A decision support system for lower back pain diagnosis: Uncertainty management and clinical evaluations. *DECISION SUPPORT SYSTEMS*. 2006;42(2):1152-1169. doi:10.1016/j.dss.2005.10.007
